# Sleep disruption from inhalation of biomass smoke: a basis for coincident hypertension?

**DOI:** 10.1186/s12989-025-00650-9

**Published:** 2025-12-11

**Authors:** K. M. Rentschler, W. Kyle Martin, W. Oshiro, M. C. Schladweiler, T. W. Jackson, W. E. Cascio, U. P. Kodavanti, P. A. Evansky, C. Lambright, R. Strader, J. Conley, W. Williams, D. Freeborn, C. N. Miller, R. Grindstaff, M. Monsees, A. A. Lewis, D. W. Herr, P. R. S. Kodavanti, M. S. Hazari, A. K. Farraj

**Affiliations:** 1 Oak Ridge Institute for Science and Education, Oak Ridge, TN 37830 USA; 2 Center for Public Health and Environmental Assessment, Office of Research and Development, US Environmental Protection Agency, 109 T.W. Alexander Drive, B105-02, Research Triangle Park, NC 27711 USA

**Keywords:** Wildfires, Biomass smoke, Cardiovascular health, Sleep, Blood pressure, Eucalyptus, Electroencephalogram, Autonomic nervous system, Air pollution

## Abstract

**Background:**

Exposure to air pollution including contemporary sources like wildland fire smoke worsens cardiovascular outcomes. Although several mechanisms for these effects have been postulated, one underexplored impact of inhaled air pollution that may mediate adverse health outcomes is sleep disruption, which is an independent risk factor for cardiovascular morbidity and a trigger of multiple biological pathways linked to disease. The purpose of this study was to determine whether cardiovascular responses to air pollution, especially excursions in blood pressure, are associated with contemporaneous changes in sleep status. Three-month old male and female Sprague Dawley rats were implanted with radiotelemetry devices that simultaneously measured aortic blood pressure and the electroencephalogram (EEG), which was used to quantify sleep quality and depth. Heart rate variability (HRV), an indirect measure of autonomic tone, and blood pressure variability (BPV) were assessed from the blood pressure signal. Rats were monitored before, during and after a single 1-hour whole body inhalation exposure to filtered air or tube furnace-generated eucalyptus smoke (632–904 µg/m^3^ fine particulate matter (PM_2.5_; ≤ 2.5 microns in aerodynamic diameter)), a key wildland fire-linked air pollution source.

**Results:**

Smoke exposure caused increases in heart rate, blood pressure, BPV, and HRV markers of sympathetic tone and concomitant disruption in several sleep parameters including slow-wave and paradoxical sleep, and wake duration to varying degrees in male and female rats relative to sex-matched filtered air controls during exposure.After exposure, smoke caused decreases in cardiovascular function and sympathetic tone that again varied by sex, although both males and females had rebound increases in sleep drive. Finally, although there were some minor sex differences, the cardiovascular and sleep responses in the smoke groups were largely more strongly correlated with one another and with HRV markers of sympathetic tone relative to responses in the respective filtered air groups.

**Conclusions:**

These findings suggest that some of the cardiovascular responses to air pollution, including hypertension, may be related to perturbations in sleep and associated changes in autonomic tone.

**Supplementary Information:**

The online version contains supplementary material available at 10.1186/s12989-025-00650-9.

## Background

The growing evidence linking wildfire smoke, an increasingly year-round and ubiquitous source of air pollution, to worsening cardiovascular health [1–3], has spurred global efforts to identify the etiological basis for disease. Previous studies have linked the adverse cardiovascular responses to inhaled air pollution in part to alterations in normal biochemical and physiological processes such as blood oxygenation [4], metabolism [6, 7], kidney function [8] and immunity [9]. Recent findings indicate that exposure to air pollution also disrupts sleep [10–13], a major determinant of health and well-being during which restorative endocrine and immune processes take place that are essential for resiliency to day-to-day stressors [14–17]. Perturbations in sleep increase risk for cardiovascular diseases such as atherosclerosis, hypertension and clinical cardiac events (e.g., myocardial infarction) and may underlie some of the cardiovascular effects of air pollution [18–28]. In fact, recent evidence suggests that circadian disruption partially mediates the association between PM_2.5_ and cardiovascular disease [29], although the extent to which specific cardiovascular effects of inhaled air pollution are related to sleep disruption is unclear.

Among the most prominent impacts of inadequate sleep, including irregular sleep [30], insomnia [31], and even one night of poor sleep [32], is daytime hypertension, characterized by elevated blood pressure during wake hours. Air pollution inhalation, including short- and long-term exposure to PM [33–35], has also been linked with daytime hypertension, although the relative proportion of these effects attributable to altered sleep status is unclear. Disrupted sleep also causes nocturnal hypertension, which is characterized by blunted blood pressure dipping during sleep and is a stronger predictor of cardiovascular mortality than daytime blood pressure [36–38]. Short-term exposures to ambient PM_10_ (coarse particulate matter; ≤ 10 microns in aerodynamic diameter) in people [39], and the combustion byproduct benzo[a]pyrene in rats [40], were also associated with non-dipping nocturnal blood pressure, with effects of the former attributed to impaired circadian handling of sodium. Although the mechanisms for these effects are unclear, sleeplessness has been linked with stress hormone release, autonomic imbalance, endothelial dysfunction, oxidative stress, and inflammation [15, 41–44], phenomena also linked with exposure to air pollution [45–49].

The linkage of air pollution exposure with poor sleep has largely been gleaned previously from human studies that documented self-reported sleep status [50] rather than objective measures such as polysomnography and sleep studies in general lacked time-matched cardiovascular measures including blood pressure. Rodent studies that demonstrated changes in blood pressure with air pollution exposure lacked assessments of sleep and early studies demonstrating the impacts of ozone on sleep were limited to measures of heart rate in tethered rats [51, 52]. The purpose of this study was to, for the first time, examine the impacts of controlled exposure to air pollution on concurrent measures of sleep and blood pressure in unrestrained rats using implantable telemetry with dual measures of the electroencephalogram (EEG) and aortic blood pressure. We hypothesized that a single exposure to air pollution would increase blood pressure and cause contemporaneous disruption in one or more aspects of sleep. Male and female rats implanted with radiotelemetry devices capable of simultaneously measuring the EEG , to quantify sleep quality and architecture, and aortic blood pressure, were exposed once to eucalyptus smoke, a key wildfire air pollution source in the United States and Australia linked to adverse cardiopulmonary outcomes [53, 54]. Sleep and cardiovascular responses were related to heart rate variability (HRV) indicators of autonomic tone, blood pressure variability (BPV) , pulmonary and systemic indicators of inflammation and injury, and circadian rhythm gene expression in the hypothalamus.

## Methods

### Animals

Female and male Sprague Dawley rats, 10–12 weeks of age, were housed one rat per cage in polycarbonate cages and maintained on a 12 h light/dark cycle at approximately 22° C and 50% relative humidity in our Association for Assessment and Accreditation of Laboratory Animal Care-approved facility and acclimated for a minimum of one week before exposures. Sprague Dawley rats were selected because they are an outbred strain [55], and we previously demonstrated that they elicit robust cardiovascular responses to biomas smoke [56, 57]. All animals received standard (5001) Purina pellet rat chow (Brentwood, MO) and water ad libitum. The Institutional Animal Care and Use Committee of the U.S. Environmental Protection Agency (U.S. EPA) approved all protocols.

### Experimental design

**Fig. 1:**
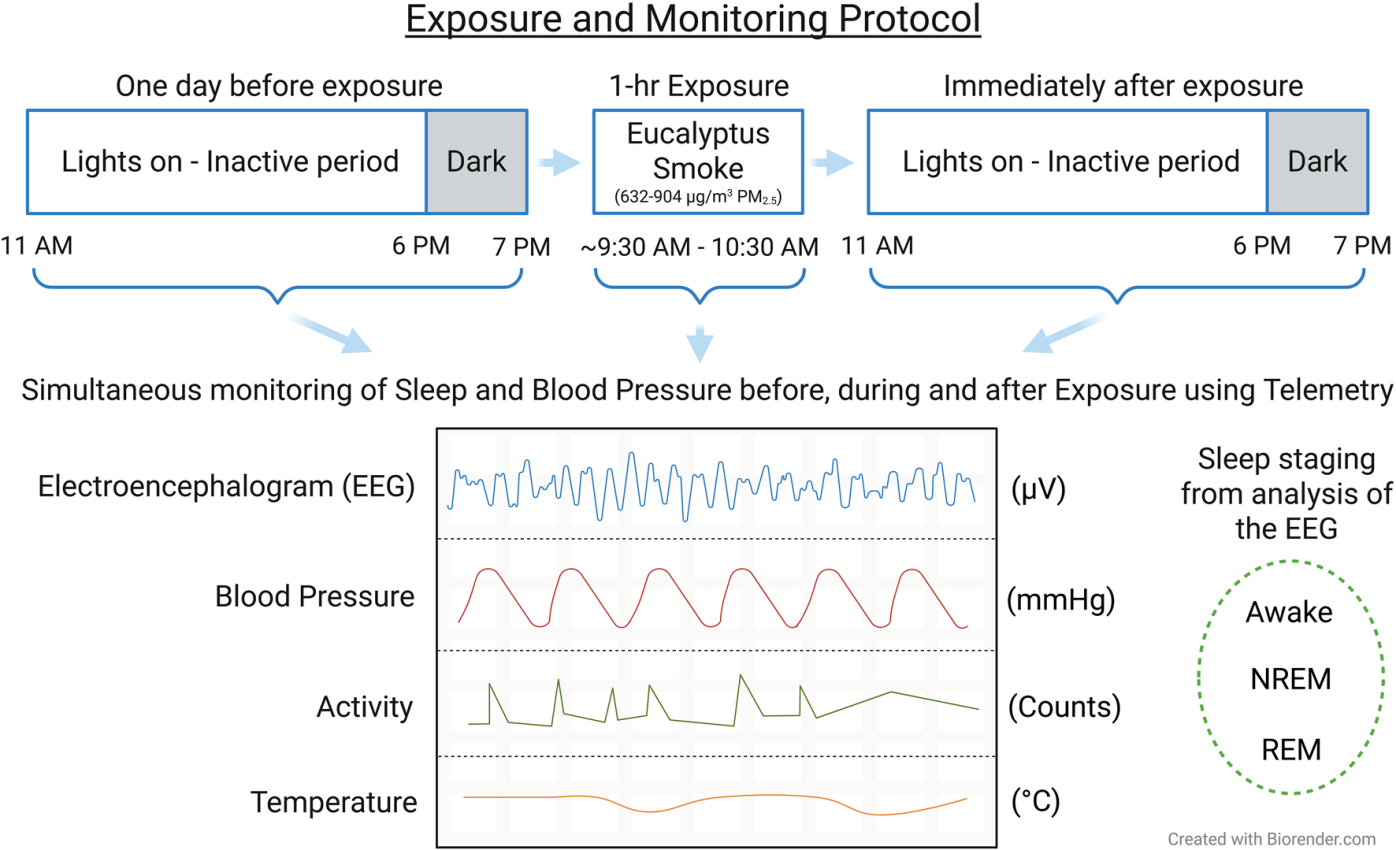
Exposure and Monitoring Protocol. Rats were implanted with a telemetry device capable of simultaneously monitoring the electroencephalogram, aortic blood pressure, activity and core body temperature. Sleep, blood pressure, activity, and core body temperature were recorded in male and female Sprague Dawley rats prior to (8 h; 11:00 AM to 7:00 PM), during (1 h; ~9:30 AM to 10:30 AM), and after (8 h; 11:00 AM to 7:00 PM) a single 1-hour exposure to filtered air or eucalyptus smoke. The EEG was used to analyze sleep quality and architecture and determine sleep staging. The blood pressure trace was used to analyze blood pressure and derive heart rate and heart rate variability determinations

The present study consisted of two cohorts of rats, each of which had four groups of rats: (1) Female-Filtered Air, (2) Female-Smoke, (3) Male-Filtered Air, and (4) Male-Smoke. Cohort 1 rats (*n* = 8/group) were exposed for 1-hr to filtered air or eucalyptus smoke and then euthanized one day later for collection and assessment of biological/tissue responses after exposure. Cohort 2 rats (*n* = 8/group) were implanted with telemeters for monitoring the electroencephalogram (EEG), blood pressure (BP), heart rate (HR; from blood pressure signal), and body temperature and then approximately two weeks after surgical recovery, underwent a single 1-hr exposure to filtered air or eucalyptus smoke as outlined in Fig. 1. A single 1-hour exposure was selected for two reasons: (1) we previously demonstrated that a single 1-hr exposure to biomass smoke increases blood pressure in rats [56, 57] consistent with the acute increases in blood pressure reported with short-term exposure to other sources of air pollution in people [58], and (2) the glass tube furnace system, described below, was designed such that the furnace moves from one end of the glass tube to the other, burning its contents in approximately 1 h. The target smoke concentration based on PM_2.5_ levels was 700 µg/m3, which is comparable to the nearly 500 µg/m3 peak concentration documented during the recent January 2025 Los Angeles, California fires [59], a significant vegetation source of which was the eucalyptus tree [60]. Group size was based on results from our previous study that examined the impact of sleep disruption on responsiveness to air pollution [56]. Note: one exposure consisting of 4 rats in the female smoke group in Cohort 1 (i.e., non-telemeter group) failed to produce any measurable PM. To address this, we exposed two available extra female rats to eucalyptus smoke. Thus, for all non-physiological endpoints in the female smoke group, the ‘n’ size was either 5 or 6, except for the gene expression data, which was limited to an ‘n’ of 4.

### Telemeter implantation

Animals were anesthetized with ketamine/xylazine (80 mg/ml ketamine HCl and 12 mg/ml xylazine HCl; Sigma Chemical Co., St. Louis, MO), and implanted with radio-telemeters that transmit EEG, aortic blood pressure, heart rate, locomotor activity and core body temperature (model HD-S11, Data Sciences International, St. Paul, MN) at Charles River Laboratories (Raleigh, NC). The pressure catheter was inserted and secured in the descending aorta, with the insertion point located just proximal to the renal artery. The EEG leads were passed through the abdominal wall and tunneled subcutaneously to the cranium, then placed into predrilled burr holes after a midline incision was made at the top of the head. The negative (-) lead was placed 2.0 mm anterior and 1.5 mm lateral to Bregma while the positive (+) lead was placed 7.0 mm posterior and 1.5 mm lateral to Bregma on the contralateral side.

### Radiotelemetry of physiological parameters

Radiotelemetry was used to monitor the EEG, BP, HR, temperature and activity in conscious, unrestrained rats. Data were recorded continuously during exposure and for four minutes every ten minutes while in their home cages before and after exposure. EEG, arterial BP (systolic and diastolic blood pressure (SBP and DBP), pulse pressure (PP; the difference between systolic and diastolic blood pressure), mean arterial pressure (MAP)) and HR were calculated automatically by software (Ponemah; Data Sciences International, Inc) from data digitally sampled at 1000 Hz.

### Acclimation to exposure chambers and Eucalyptus smoke concentrations

All animals were acclimated three times in 10 min increments to full-body inhalation chambers at room temperature. Final acclimations occurred at least 24 h before exposure. We used eucalyptus wood which was purchased as writing pen blanks (rectangles at 0.75 inches square by 6 in long; Woodworkers Source Arizona). This wood was processed through a gasoline powered wood shredder (Echo bearcat model number SC3206). The shredder was cleaned between uses so no cross contamination would occur. Animals were exposed once for a duration of 1 h to eucalyptus smoke generated using an automated control tube furnace system (as described below).

### Tube furnace exposure system and PM and gas monitoring and sampling

Eucalyptus smoke was generated using an automated quartz-tube furnace system that burned the eucalyptus wood material under flaming (640 °C) combustion conditions. An automated mass flow controller (Mass-Flo, MKS Instrument, Inc., Andover, MA) based on a proportional-integral-derivative (PID) feedback loop was incorporated into the system to precisely control smoke concentration. Eucalyptus smoke (2 L/min) generated from the tube furnace system was diluted with filtered air (∼3 and ∼60 L/min for 1st and 2nd dilution air, respectively) and then delivered to a whole-body inhalation chamber as described previously [57]. The smoke concentration was monitored continuously and adjusted by the PID feedback control loop linked to a continuous PM monitor in the chamber linked to an exhaust flow control valve in a smoke inlet line. The adjustment was made immediately after a change in PM_2.5_ concentration in the chamber was detected to maintain PM_2.5_ concentration close to its set point (< 10% of the target set point). The smoke in the chamber was maintained at a temperature of ∼72° F, and ∼40% relative humidity, controlled by a humidifier. A pressure gauge (Magnehelic, Dwyer Instruments Inc., Michigan City, IN) was placed in the chamber to ensure constant pressure throughout the inhalation exposure. Carbon dioxide (CO_2_) and carbon monoxide (CO) levels were monitored using a non-dispersive infrared analyzer (Model: 602 CO/CO_2_; CAI Inc., Orange, CA). PM_2.5_ was collected on a glass-fiber filter installed in an exhaust line of the inhalation chamber to determine mean PM_2.5_ concentrations gravimetrically by weighing the filter before and after inhalation exposure. Real-time measurements of smoke properties and engineering parameters (i.e., temperature, RH, static pressure, and flow rate) were continuously monitored and recorded using data acquisition software (Dasylab version 13.0, National Instruments, Austin, TX).

### Sleep data acquisition and analysis

EEG data and home cage activity were recorded using Ponemah 6.10 software (Data Sciences International, Inc.). The EEG signal data was digitized at a continuous sampling rate of 1000 Hz. EEG data were scored with NeuroScore 3.4 software (Data Sciences International, Inc.). Data were scored within 10 s epochs and vigilance states were determined as wake (low amplitude, high-frequency EEG), rapid eye movement (REM; also known as paradoxical sleep and characterized by rapid eye movement with low amplitude, high theta frequency EEG and an absence of activity levels) or non-rapid eye movement sleep (NREM; also known as slow-wave sleep and characterized by high amplitude, low frequency EEG and absent activity levels). Power band visualizations and measures of home cage activity were used to confirm vigilance states with the absence of the electromyogram. If theta power bands were most prominent within the 10s epoch, and were preceded by NREM sleep, the EEG was scored as REM sleep by the visual scorer to distinguish REM sleep from quiescent wakefulness. Transitions between vigilance states were scored if two consecutive 10 s epochs of a new sleep state were noted. EEG spectral power data were calculated using discrete fast Fourier transform for each 10 s epoch for delta (0.5–4 Hz), theta (4–8 Hz), alpha (8–12 Hz), sigma (12–16 Hz) and beta (16–20 Hz) oscillations. Spectral power data were normalized for each vigilance state: wake, REM and NREM across the entire power spectrum (0.5–20 Hz), and a focused analysis for alpha, delta and theta frequencies were conducted for wake, NREM and REM, respectively. The following sleep parameters were measured for each vigilance state: total duration, average bout duration, and bout number. For pre- and post-exposure data, percent change calculations were used to display the impact of the exposure on vigilance state parameters. When an animal had zero REM bouts or REM duration during the exposure period, the animal was excluded from the analysis, as REM was not present.

### Heart rate variability analysis

The blood pressure signal was used to derive HRV because the electrocardiogram (ECG) was not measured. HRV derived from analysis of the blood pressure waveform has been previously shown to generate data in good agreement with HRV measures derived from analysis of RR waves of the ECG [61, 62]. The variability in the inter-beat interval (IBI), which was calculated as the time in milliseconds between successive peaks in the systolic pressure wave (with artifacts and abnormal waveforms excluded), was used to generate time-domain measures, including standard deviation of the RR interval (SDNN) and square root of the mean of squared differences of adjacent RR intervals (RMSSD). Analysis also provided frequency-domain parameters, including very low frequency (vLF), low frequency (LF), high frequency (HF), and the ratio of these two frequency domains (LF/HF), as well as normalized LF (nLF) and HF (nHF), which are each derived by dividing LF or HF by the total of LF and HF. For frequency-domain analysis, the signal was analyzed with a Hamming window for segment lengths of 512 samples with 50% overlapping. Within Ponemah, the settings for variability analysis in the frequency domain were vLF (0.05–0.25), LF (0.25–1.0), and HF (1.0–3.0) for the “rat bin set.” HRV was analyzed for the entirety of the exposure period as well as each hour during the pre- and post-exposure periods. HRV during exposure is reported as averages per 5-minute period. Post-exposure effects are reported as percent change from pre-exposure for each hour post-exposure.

### Blood pressure variability analysis

Blood pressure variability (BPV) was analyzed with an open- source R package called bp [63]. This package calculates several metrics of dispersion of blood pressure, including average real variability (ARV), coefficient of variation (CV), standard deviation (SD) and successive variation (SV). The exposure period as well as each hour during the pre- and post-exposure periods were analyzed. During exposure, BPV was calculated as an average over the 60-minute period of exposure (i.e., from 60 one-minute averages during exposure). During the pre-and post-exposure periods, BPV values for each hour were derived from 24 one-minute averages per hour and are reported as percent change from pre-exposure.

### Necropsy and tissue collection

Cohort 1 rats were euthanized via intraperitoneal injection of 1 ml/kg pentobarbital (Fatal-Plus, Dearborn, MI) diluted 1:1 approximating 200 mg/ml one day after exposure. When animals were completely non-responsive to hind paw pinch, blood was collected through the abdominal aorta in serum separator tubes and ethylenediaminetetraacetic acid (EDTA) tubes, which were inverted and placed on ice. Before spinning the EDTA tubes, complete blood cell counts (CBCs) were determined. Red blood cells (RBCs), white blood cells (WBCs), red cell distribution width (RDW%), hematocrit (HCT), mean corpuscular volume (MCV), hemoglobin (HB), mean corpuscular hemoglobin (MCH), mean corpuscular hemoglobin concentration (MCHC), platelets (PLTs), plateletcrit (PCT) and mean platelet volume (MPV) were measured utilizing a Beckman-Coulter AcT blood analyzer (Beckman-Coulter Inc., Fullerton, CA). The serum and EDTA tubes were then centrifuged at 1500 x g for 10 min and serum and plasma samples were stored at − 80 °C until further analysis. The hypothalamus was excised, snap frozen and stored at − 80 °C until further analysis.

### Measures of systemic markers and hormones

Several serum factors were measured using the Konelab Arena 30 Clinical Chemistry Analyzer (Thermo Clinical LabSystems, Espoo, Finland) including total cholesterol (TC), triglycerides (TG), creatine kinase (CK), alkaline phosphatase (ALP), alanine amino-transferase (ALT), glucose (TECO Diagnostics, Anaheim, CA), high-density lipoprotein (HDL) and low-density lipoprotein (LDL), (Sekisui Diagnostics, PE, Canada), free fatty acids (FFA; Cell Biolabs, Inc., San Diego, CA), complement C3 and complement C4 (Kamiya Biomedical, Seattle, WA), and angiotensin-converting enzyme (ACE) ( Trinity Biotech USA, Jamestown, NY). The serum was also assayed for the cytokines interferon(IFN)-γ, interleukin(IL)-1β, IL-4, IL-5, IL-6, IL-10, IL-13, keratinocyte chemoattractant/growth-related oncogene (KC-GRO), and tumor necrosis factor-α (V-PLEX Pro-inflammatory Pane 2 Rat kit; Meso Scale Discovery, Rockville, MD), and for the hormones C-peptide, glucagon-like peptide-1 (GLP-1), glucagon, insulin, leptin, and peptide YY (PYY) (U-Plex Diabetes Combo 1 (rat); Meso Scale Discovery, Rockville, MD).

### Bronchoalveolar lavage fluid (BALF) collection and analysis

The tracheae of Cohort 1 rats were cannulated at necropsy and then a suture was used to tie the left lung. The right lung was lavaged using Ca2+/Mg2 + free phosphate buffered saline (pH 7.4) equal to 28 mL/kg body weight (total lung capacity) × 0.6 (right lung is ~ 60% of total lung weight). Three in-and-out washes were performed using the same aliquot of buffer, and BALF was transferred to ice until further processing. BALF samples were processed to determine total cell counts and cell differentials (Z1 Beckman-Coulter Counter, Miami, FL). Cell-free BALF samples were used to measure markers of injury using the Konelab Chemistry Analyzer. Separate kits were used to measure total protein (Coomassie Plus Protein Assay Kit; Pierce Biotechnology, Inc., Rockford, IL), albumin (Sekisui Diagnostics, PE, Canada), lactate dehydrogenase (LDH) activity, γ-glutamyl transferase (GGT) activity (Thermo Fisher Diagnostics, Middletown, VA), and β-N-Acetylglucosaminidase (NAG) activity (Roche Diagnostics, Indianapolis, IN).

### RNA isolation and measurement of gene expression in the hypothalamus

Samples of hypothalamus tissue (~ 20–40 mg) were placed into polypropylene microcentrifuge tubes containing 500 µL TRIzol Reagent (Invitrogen, Carlsbad, CA, USA) on ice, then individually homogenized using a Bullet Blender (Next Advance, Troy, NY, USA) with 1 mm zirconium oxide beads and stored at − 80 °C. RNA extraction was conducted according to TRIzol Reagent manufacturer specifications using chloroform and isopropanol. Following extraction, RNA was purified using GeneJet RNA clean up and concentration kit (Cat no. K08401; Thermo Fisher Scientific, Waltham, MA, USA). RNA concentration and purity (260:280 ratio ≥ 1.8) were determined with a NanoDrop 2000 spectrophotometer (Thermo Scientific). cDNA was synthesized from purified RNA using the RT2 First Strand kit (Qiagen, Hilden, Germany) and gene expression was assessed using reverse transcriptase real-time quantitative polymerase chain reaction (RT-qPCR).

Hypothalamic tissue was evaluated using the RT2 Profiler PCR Array for Rat Circadian Rhythm targets by Qiagen (Cat. no. 330231 PARN-153Z), which contains 84 target genes relevant to the circadian clock, casein kinases, cAMP response element-binding protein (CREB) signaling, light sensing proteins, and circadian regulated transcription factors. PCR reactions were run using RT2 SYBR Green qPCR Master Mix (Qiagen) on a CFX96 Touch Real-Time Detection System (Bio-Rad, Hercules, CA, USA).

### Statistical analysis

Data are reported as bar graphs with all individual data points shown, or line graphs as simple correlations, or plotted points with error bars representing means ± SEM. GraphPad Software (version 10.4.1, San Diego, CA) was used for all visualizations, and GraphPad or R (version 4.4.0) were used for all statistical analyses. For all data, group comparisons were limited to within sex and not made across sex because of the baseline differences in various cardiovascular parameters evident in these animals. Given that we previously demonstrated that exposure to various sources of air pollution including peat smoke [57], ozone [64], and diesel exhaust [65] causes short-term often minute-by-minute and hour-by-hour changes in blood pressure, heart rate, and cardiac arrhythmia among other effects, we analyzed and present exposure data by effects overall and by 1-minute, 5 min, 30 min, and/or 60-minute averages, while post-exposure data was presented as average percent change for each hour post-exposure relative to 1 h averages pre-exposure or the percent change for the entire post-exposure period. For all time course cardiovascular physiology, sleep duration, and HRV and BPV analyses, data were analyzed using a generalized linear mixed model with eucalyptus smoke (filtered air vs. eucalyptus smoke) and exposure time and multiplicative fixed factors and unique subject identifier as random factors in the analyses with repeated measures. All linear models were performed using the glmmTMB package [66], with pairwise comparisons performed using estimated marginal means from the emmeans package in R [67]. A p-value < 0.05 was set as a threshold for statistical significance for both familywise and pairwise comparisons. The linear mixed model was used to assess overall effects and for comparisons at specific intervals in time. For sleep spectral data, variables were assessed with repeated measures two-way ANOVA with Sidak’s post hoc test and multiplicity-adjusted p values with p values < 0.05 considered statistically significant. For comparisons of group means (filtered air vs. smoke) within sex during specific snapshots in time (i.e., 30-min averages during exposure, the entire exposure period and the entire post-exposure period, biological data), the data were analyzed using parametric unpaired t tests. Data that did not meet the normality assumption were tested using the nonparametric Kruskal-Wallis test with Dunn’s multiple comparisons post hoc test. Correlations between variables were determined using simple linear regression for determination of r^2^ values with p value < 0.05 considered statistically significant.

RT-qPCR gene expression data were analyzed using the comparative cycle threshold (CT) method. Melt curve analyses were conducted for all genes in each treatment group, and any well not displaying a clear peak was assigned a CT value of 35. Delta CT values were calculated using the Eq. 2-∆∆CT and normalized to the mean CT value of at least 2 housekeeping genes for each tissue and gene array. We selected housekeeping genes that did not display significant treatment effect of exposure (*B2m*,* Hprt1*,* and Ldha*). Delta CT values were then converted to fold-induction by dividing the treated replicate delta CT by the mean delta CT of the control replicates for each gene. Fold induction values were log10 transformed prior to ANOVA to correct for heterogeneity of variance. ANOVA p-values were then corrected using False Discovery Rate (FDR) adjustment in Prism (“Two-stage step-up method of Benjamini, Krieger, and Yekutieli”; Desired FDR = 5%).

## Results

### Characterization of Eucalyptus smoke

Gas and particle concentrations for all exposures are indicated in Table 1. The average eucalyptus smoke exposure concentration during the female exposures was 904 µg/m^3^, whereas the average concentration for the male exposures was 632 µg/m^3^ (Table 1). The average particle size for the exposures in the present study ranged from 0.391 to 0.399 microns. Although below and above the target concentration of 700 µg/m3, these concentrations are still comparable to peak concentrations documented during recent wildfire events [59].

**Table 1:** Characteristics of Eucalyptus smoke in the inhalation exposure chambers

	Female Exposures		Male Exposures	
	Filtered Air	Eucalyptus Smoke	Filtered Air	Eucalyptus Smoke
PM_2.5_ (µg/m^3^)	3.00 (0–6) ± 4.24	904 (823–985) ± 115	0.00 ± 0.00	632 (532–732) ± 141
PM size	---	0.399 ± 1.30^b^	---	0.391 ± 1.40
CO (ppm)	1.10 ± 0.00	48.0 ± 0.00	1.12 (1.1–1.2) ± 0.07	45.0 (44–45) ± 0.71
CO_2_ (ppm)	0.10 ± 0.00	1032 (932–1131) ± 141	0.10 ± 0.00	1372 (1349–1394) ± 32.0
Chamber Temp. [F]	74.2 (74.0–74.4) ± 0.28	73.0 (72–74) ± 1.41	73.3 (72.3–74.2) ± 1.34	74.0 ± 0.00
Chamber RH [%]	46.5 (46–47) ± 0.71	46.0 ± 0.00	45.0 (44–45) ± 0.71	46.0 ± 0.00

Exposure concentrations were averaged over 2 days for the females and 2 days for the males for exposures conducted in January 2022. There was an error in the gravimetric measures of PM concentration for one of the filtered air exposures for the males, so the value indicated for PM is for one day; the remaining values for the male filtered air exposure are averages of two days of exposure. *PM*_*2.5*_ particulate matter < 2.5 microns in aerodynamic diameter, *CO* carbon monoxide, *CO*_*2*_ carbon dioxide, *RH* relative humidity, Unfortunately, there were no working instruments available for measurement of NO/NO2/NOx for this project, but our previous eucalyptus smoke study in this PM_2.5_ concentration range yielded the following concentrations: NO – 0.272 ppm, NO2–0.428 ppm, and NOx 0.388 ppm (Martin et al. 2023)

### Heart Rate, blood pressure and temperature during and after exposure

**Fig. 2:**
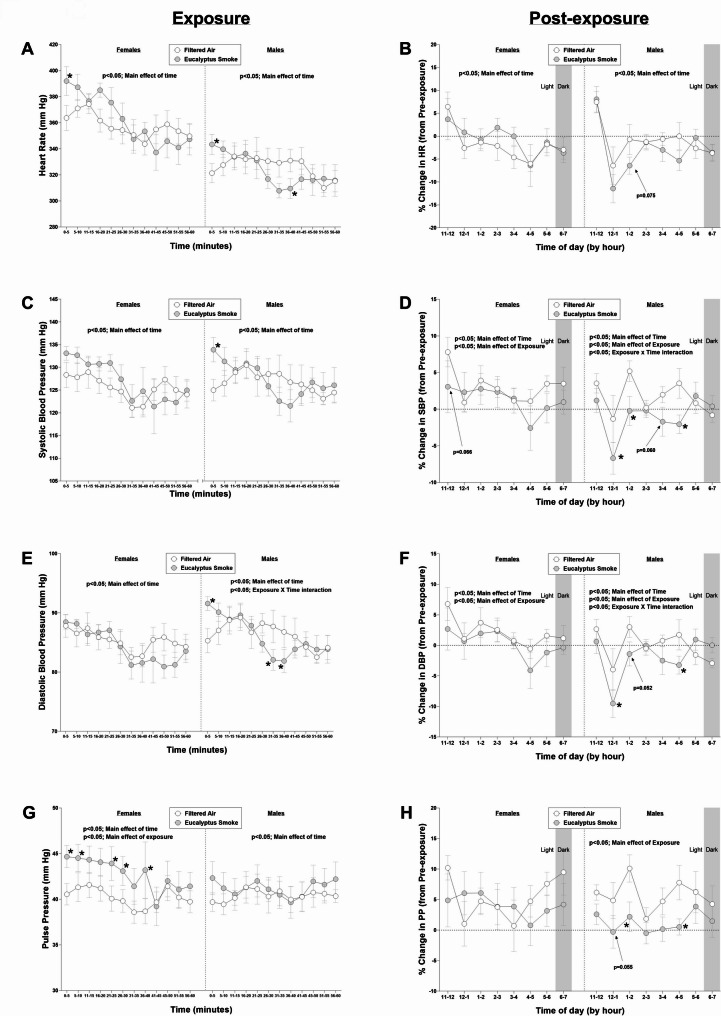
Eucalyptus smoke exposure changes cardiovascular parameters both during and following exposure. Cardiovascular physiology was recorded using implantable telemetry in male and female Sprague Dawley rats to measure cardiovascular parameters prior to (8 hrs; 11:00 AM to 7:00 PM), during (1 hr; ~9:30 AM to 10:30 AM), and after (8 hrs; 11:00 AM to 7:00 PM) filtered air or eucalyptus smoke exposure. Data were separated by sex. The following measurements were collected: (a) heart rate measurements during exposure (b) percent change in heart rate post-exposure (relative to pre-exposure) (c) systolic blood pressure during exposure (d) percent change in systolic blood pressure post-exposure (relative to pre-exposure) (e) diastolic blood pressure during exposure (f) percent change in diastolic blood pressure post-exposure (relative to pre-exposure) (g) pulse pressure during exposure and (h) percent change in pulse pressure post-exposure (relative to pre-exposure). Data are mean ± SEM and were analyzed by a generalized linear mixed model. Data during exposure are reported in 5-min intervals; percent change data after exposure are reported in 1-hr intervals. *p < 0.05. n = 8 per group

Figures 2 and S1 show five-minute and one-minute averages of cardiovascular physiology data, respectively, recorded during the entire exposure period. With respect to overall effects in one-minute data during exposure, there was a significant overall effect of exposure on HR (*p* < 0.0001) and PP (*p* < 0.0001) in females and on PP (*p* < 0.05) in males. There was also a significant overall effect of time on HR (*p* < 0.0001), SBP (*p* < 0.0001), DBP (*p* < 0.0001), PP (*p* < 0.0001), and MAP (*p* < 0.0001) in females and on HR (*p* < 0.0001), SBP (*p* < 0.0001), DBP (*p* < 0.0001), and MAP (*p* < 0.0001) in males. There were also significant interactions between exposure and time in SBP (*p* < 0.0001), DBP (*p* < 0.0001), and MAP (*p* < 0.05) in males. With respect to overall effects in five-minute data during exposure, there was a significant overall effect of exposure on PP (*p* < 0.0001) and a tendency towards a significant overall effect of exposure on SBP (*p* = 0.0659) in females. There was also a significant effect of time on HR (*p* < 0.0001), SBP (*p* < 0.05), DBP (*p* < 0.05), PP (*p* < 0.05), and MAP (*p* < 0.05) in females and HR (*p* < 0.05), SBP (*p* < 0.05), DBP (*p* < 0.0001), and MAP (*p* < 0.05) in males. There were also significant interactions between exposure and time in DBP (*p* < 0.05), and MAP (*p* < 0.05) in males.

When averaged over 5-minutes, smoke exposure significantly increased HR at minutes 0–5 (*p* < 0.05; Fig. 2A) and caused tendencies towards significant increases at minutes 16-20 (*p* =0.0799) and 21-25 (*p* = 0.0795) in female rats. In one-minute data in female rats, smoke exposure significantly increased HR at multiple points during the first half of the exposure period (minutes 2, 5, 6, 17, 18, 21, and 24; *p* < 0.05), and caused tendencies towards significant increases at minutes 1 (*p* = 0.0594), 3 (*p* = 0.0509), 22 (*p* = 0.0558), and 23 (*p* = 0.0615) relative to female rats exposed to filtered air (*p* < 0.05; Figure S1A). When averaged over 5-minutes in male rats, smoke exposure significantly increased HR at minutes 0–5 (*p* < 0.05) and significantly decreased HR at minutes 36-40 (*p < 0.05*; Fig. 2A). In one-minute data in male rats, smoke exposure significantly increased HR at minutes 4 and 5 (*p* < 0.05). By the half-way point of the exposure regimen, the response to smoke exposure had shifted in male rats such that they now had significant decreases in HR relative to male rats exposed to filtered air (at minutes 34, 39, 40, and 43; *p* < 0.05; Figure S1A).

When averaged over 5-minutes in female rats, smoke exposure had no significant effect on SBP (Fig. 2C) and DBP (Fig. 2E). In one-minute data in female rats, smoke exposure significantly increased SBP at multiple points during the first half of the exposure period (minutes 2, 6, 20, 21, and 23; *p* < 0.05; Figure S1B) but had no effect on DBP (Figure S1-C). When averaged over 5-minutes in male rats, smoke exposure caused significant increases in SBP and DBP during minutes 0–5 relative to filtered air controls (*p* < 0.05; Fig. 2C) and significant decreases in DBP at minutes 31–35 and 36–40 (*p* < 0.05; Fig. 2E). In one-minute data in male rats, smoke exposure significantly increased SBP and DBP during 5 of the first 10 min of exposure (minutes 3 through 7; *p* < 0.05; Figure S1B to S1C) relative to male rats exposed to filtered air. Smoke exposure also caused a significant decrease in SBP at the 29-minute mark (*p* < 0.05; Figure S1B) and significant decreases in DBP at several periods (minutes 29, 33–35, and 37–38; *p* < 0.05; Figure S1C) relative to males exposed to filtered air like the pattern observed for heart rate. In five-minute data, smoke exposure increased PP at minutes 0–5, 6–10, 21–25, 26-30, and 36-40 in female rats (*p* < 0.05) and caused tendencies towards significant increases at minutes 11-15 (*p* = 0.0609) and 16-20 (*p* = 0.0747) but had no effect in male rats (Fig. 2G). In one-minute data in female rats, smoke exposure caused significant increases in PP during nearly half of the exposure regimen (25 of the 60 min of exposure; *p* < 0.05) and tendencies towards significant increases at minutes 27 (*p* = 0.0560), and 30 (*p* = 0.0536) relative to female rats exposed to filtered air, whereas smoke exposure in male rats caused a significant increase in PP only at minute 6 relative to male rats exposed to filtered air (*p* < 0.05; Figure S1E). In one-minute data in female rats, smoke exposure caused a significant increase in MAP only at minute 6 (*p* < 0.05; Figure S1E) relative to female rats exposed to filtered air and had no significant impact when averaged over 5-minutes (Figure S2A). When averaged over 5-minutes, the impacts of smoke exposure on MAP in male rats were similar and largely concurrent with the DBP responses, where smoke exposure caused a significant increase in MAP during minutes 0–5 relative to filtered air controls and significant decreases at minutes 31–35 and 36–40 (*p* < 0.05; Figure S2-A). In one-minute data, a similar pattern of initial significant increases in MAP (minutes 3–7; *p* < 0.05) was followed by significant decreases (minutes, 29, 33–35, 37, and 42; *p* < 0.05; Figure S1C) relative to males exposed to filtered air.

With respect to overall effects after exposure, there was a significant overall effect of exposure on SBP (*p* < 0.05), DBP (*p* < 0.05), and MAP (*p* < 0.05) in females and SBP (*p* < 0.05), DBP (*p* < 0.05), PP (*p* < 0.0001) and MAP (*p* < 0.05) in males. There was also a significant overall effect of time on HR (*p* < 0.0001), SBP (*p* < 0.05), DBP (*p* < 0.0001), and MAP (*p* < 0.0001) in females and on HR (*p* < 0.0001), SBP (*p* < 0.0001), DBP (*p* < 0.0001), and MAP (*p* < 0.0001) in males. There were also significant interactions between exposure and time in SBP (*p* < 0.05), DBP (*p* < 0.05), and MAP (*p* < 0.05) in males. With respect to hourly changes (reported as % change from time-matched pre-exposure period for each hour), there was no significant effect of smoke exposure on HR in female rats relative to female rats exposed to filtered air, whereas smoke exposure in male rats caused a tendency towards a significant decrease in HR at hours 2–3 PM (*p* = 0.075; Fig. 2B) relative to male rats exposed to filtered air. Smoke exposure in female rats caused a tendency towards a significant decrease in SBP (*p* = 0.066; Fig. 2D) between 11AM and 12 PM relative to female rats exposed to filtered air. By contrast, smoke exposure in male rats caused a significant decrease in SBP (Fig. 2D), and MAP (Figure S2) at hours 12 − 1 PM, 1–2 PM, and 4–5 PM (*p* < 0.05) and caused a tendency towards a significant decrease in SBP at hours 3–4 PM (*p* = 0.060) relative to males exposed to filtered air. Smoke exposure in male rats also caused a significant decrease in DBP at hours 12 − 1 PM, and 4–5 PM and caused a tendency towards a decrease at hours 1–2 PM (Fig. 2F; *p* = 0.052). There were no significant impacts of exposure on hourly DBP in females after exposure. Smoke exposure in males caused a significant decrease in PP at hours 1–2 PM, and 4–5 PM (*p* < 0.05) and a tendency towards a significant decrease at hours 12 − 1 PM (Fig. 2H; *p* = 0.055), whereas there was no significant effect of smoke on hourly PP in females after exposure (Fig. 1H ).

Figures S2C and S2D show temperature data before and after exposure, respectively. During exposure, there was a significant overall effect of time on temperature in females (*p* < 0.0001) and males (*p* < 0.0001). There was also a significant interaction between exposure and time on temperature (*p* < 0.05) in females. Smoke exposure caused a significant decrease in core body temperature at minutes 0–5 and 51–55 and a tendency towards a significant decrease at minutes 6–10 (0.0618) and 21–25 (0.0523) in females relative to females exposed to filtered air. After exposure, there was a significant interaction between exposure and time in temperature (*p* < 0.0001) in females. There was also a significant overall effect of time (*p* < 0.0001) on temperature and a significant interaction between exposure and time (*p* < 0.05) in temperature in males.

### Polysomnography and activity during and after exposure

**Fig. 3:**
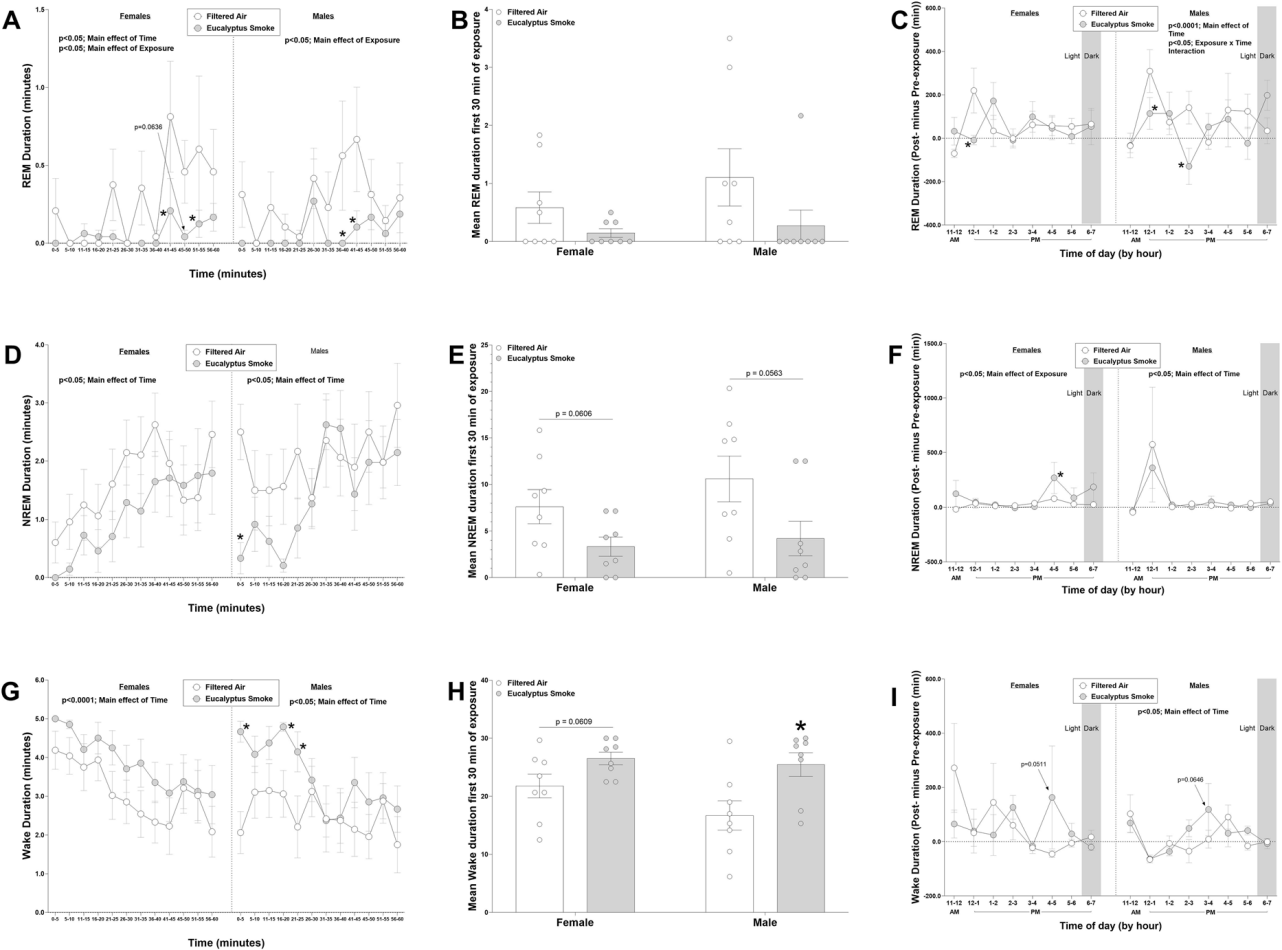
Eucalyptus smoke exposure significantly alters sleep and wake duration during exposure. Electroencephalogram (EEG) data were recorded using implantable telemetry in male and female Sprague Dawley rats to measure the polysomnography parameters rapid-eye-movement (REM) sleep, non-rapid eye movement (NREM) sleep, and wake prior to (8 h; 11:00 AM to 7:00 PM), during (1 h; 9:30 to 10:30 AM), and after (8 h; 11:00 AM to 7:00 PM) filtered air or eucalyptus smoke exposure. Data were separated by sex. The following measurements were collected: (**a**) REM duration during exposure, 5-min intervals (**b**) mean REM duration during the first 30-minutes during exposure (**c**) REM duration, post- minus pre-exposure (**d**) NREM duration during exposure, 5-min intervals (**e**) mean NREM duration during the first 30-minutes during exposure (**f**) NREM duration, post- minus- pre-exposure (**g**) wake duration during exposure, 5-min intervals (**h**) mean wake duration during the first 30-minutes during exposure and (**i**) wake duration, post- minus pre-exposure. Data are mean ± SEM and were analyzed by a generalized linear mixed model for figures (a, c, d, f, g, and I), or by unpaired Student’s t-test (figures b, e and h.). Data during exposure are reported in 5-min intervals; percent change data after exposure are reported in 1-hr intervals. *p < 0.05, **p < 0.01. *n* = 8 per group

**Fig. 4:**
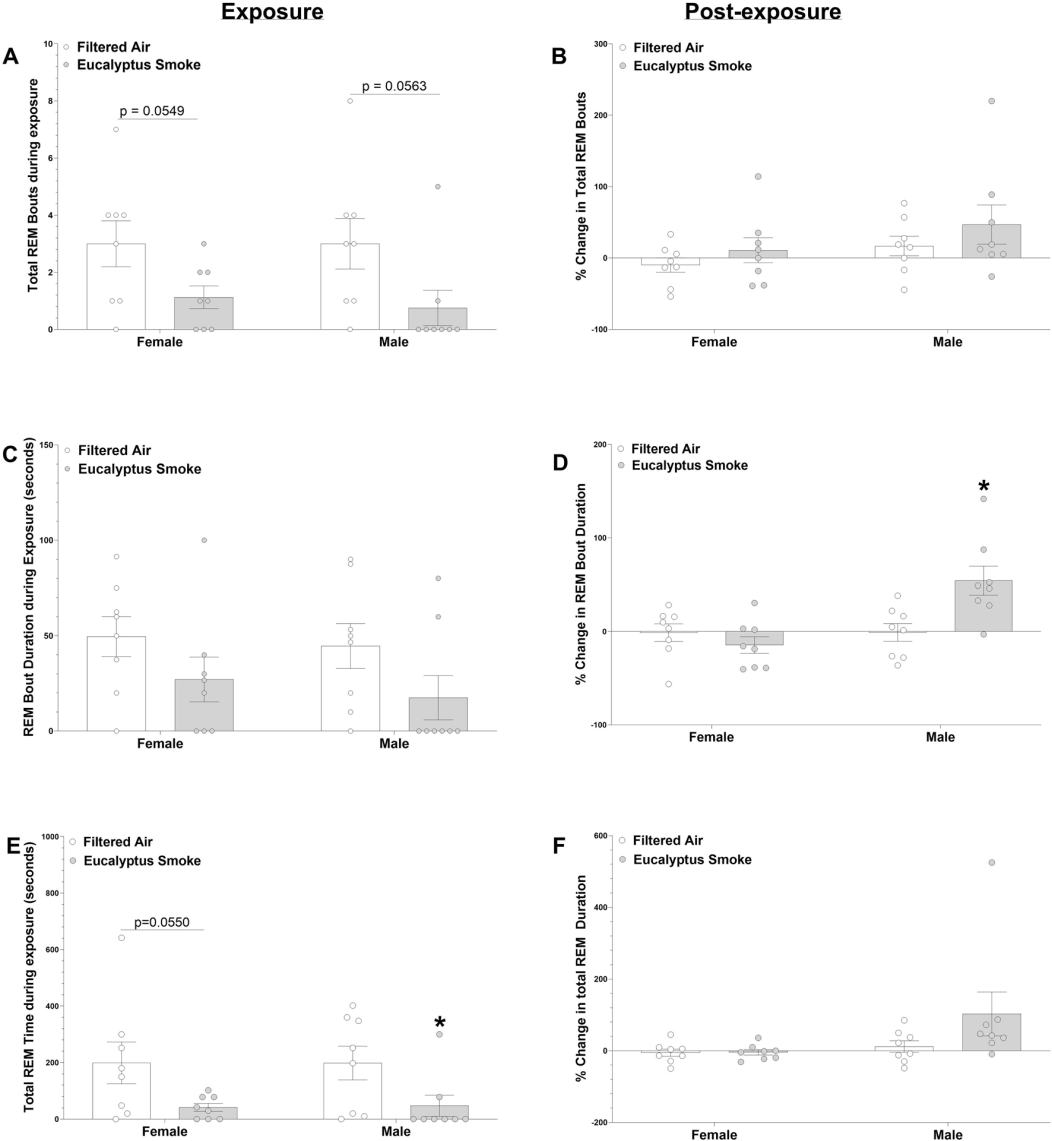
Eucalyptus smoke exposure significantly impacts rapid-eye-movement (REM) sleep in males. Electroencephalogram (EEG) data were recorded using implantable telemetry in male and female Sprague Dawley rats to measure REM sleep prior to (8 hrs; 11:00 AM to 7:00 PM), during (1 hr; 9:30 AM to 10:30 AM), and after (8 hrs; 11:00 AM to 7:00 PM) filtered air or eucalyptus smoke exposure. Data were separated by sex. The following measurements were collected: (a) Total REM bouts during exposure (b) percent change in total REM bouts post-exposure (relative to pre-exposure) over 8 hrs (c) REM bout duration during exposure (d) percent change in REM bout duration post-exposure (relative to pre-exposure) over 8 hrs (e) total REM time during 1-hr exposure and (f) percent change in total REM time post-exposure (relative to pre-exposure) over 8 hrs. Data are mean ± SEM and were analyzed by unpaired Student’s t-test. Data during exposure are reported in 5-min intervals; percent change data after exposure are reported in 1-hr intervals. *p < 0.05, **p < 0.01. n = 8 per group

Figure 3 shows REM, NREM, and Wake duration data during and after exposure. Because inhalation exposure in the present study took place during the “lights-on” period, which corresponded to the normal inactive/sleep period of rats, filtered air-exposed animals began sleeping shortly after placement in the exposure chamber., sleeping from ~ 20 to 60% of the time during each 5-minute interval of exposure. During exposure, there was a significant overall effect of exposure (*p* < 0.05) and time (*p* < 0.05) on REM duration in females (Fig. 3A), and a significant overall effect of exposure (*p* < 0.05) on REM duration in males (Fig. 3A). There was a significant overall effect of time on NREM duration in females (*p* < 0.05) and males (*p* < 0.05; Fig. 3D). There was a significant overall effect of time (*p* < 0.0001) and a tendency towards a significant overall effect of exposure (*p* = 0.0721) on Wake duration in females (Fig. 3G). There was a significant overall effect of time (*p* < 0.05) and a tendency towards a significant overall effect of exposure (*p* = 0.0593) on Wake duration in males (Fig. 3G). By time interval during exposure in female rats, smoke exposure significantly decreased REM duration during minutes 41–45 and 51–55 (*p* < 0.05) and caused a tendency towards a decrease at minutes 46–50 (*p* = 0.0636) relative to filtered air controls during exposure. In male rats, smoke exposure significantly decreased REM duration during minutes 36–40 and 41–45 relative to filtered air controls during exposure (Fig. 4A). Smoke exposure also significantly decreased NREM duration during minutes 0–5 of the exposure and increased Wake duration (*p* < 0.05) during minutes 0–5, 16–20 and 21–25 of the exposure period relative to male rats exposed to filtered air (*p* < 0.05), but caused no significant change in female rats (Fig. 4A and G). When totaling duration data for the first 30-minutes in female rats, smoke exposure caused a tendency towards a significant decrease in NREM duration (*p* = 0.0606) and increase in Wake duration (*p* = 0.0609) relative to female rats exposed to filtered air. When totaling duration data for the first 30-minutes in male rats, smoke exposure also showed a tendency towards a significant decrease in NREM duration (*p* = 0.0563) and significantly increased Wake duration (*p* < 0.05) relative to male rats exposed to filtered air (Fig. 3E and H)

After exposure, there was a significant overall effect of time (*p* < 0.0001) and a significant interaction between exposure and time (*p* < 0.05) on REM duration in males (Fig. 4C). There was a significant overall effect of exposure (*p* < 0.05) and a tendency towards a significant overall effect of time (*p* = 0.0701) on NREM duration in females (Fig. 4F). There was a significant overall effect of time (*p* < 0.05) on NREM duration in males (Fig. 4F). There was a significant overall effect of time (*p* = 0.0001) on Wake duration in males (Fig. 4F) and a tendency towards a significant overall effect of time (*p* = 0.0553) on Wake duration in females (Fig. 4I). Smoke exposure significantly decreased the % change in REM duration at hours 12 − 1 PM in female rats (Fig. 4C; *p* < 0.05) and at hours 12 − 1 PM and 2-3 PM in male rats, relative to their respective sex-matched filtered air controls (*p* < 0.05). Smoke exposure significantly increased the % change in NREM duration at hours 4–5 PM after exposure in female rats (Fig. 4F; *p* < 0.05) but caused no change in male rats. Smoke exposure also caused tendencies toward increased % change in Wake duration at hours 4–5 PM in female rats (Fig. 4I; *p* = 0.0511) and hours 3–4 PM in male rats (*p* = 0.0646).

Figure 4 shows REM bout number (Fig. 4A and B), REM bout duration (Fig. 4C and D) and total REM time (Fig. 4E and F) during and after exposure (reported as % change from time-matched pre-exposure period for each hour) while Figure S3 shows bout data for NREM and Wake. In female rats, smoke exposure caused a tendency towards a significant decrease in REM bout number (*p* = 0.0549) and a trend towards a significant decrease in total REM time (*p* = 0.055) relative to female rats exposed to filtered air during exposure, but no significant change post-exposure. In male rats, smoke exposure caused a significant decrease in total REM time during exposure (*p* < 0.05) and an increase in the % change in REM bout duration after exposure relative to the time-matched data prior to exposure (*p* < 0.01). Smoke exposure caused a tendency towards a significant decrease in REM bout number (p = 0.0563) in male rats relative to male rats exposed to filtered air during exposure but no significant change in REM bout number or REM duration post-exposure. There were also no significant effects of smoke exposure in either female or male rats in NREM or Wake bout number, duration or time (Figure S3).

Figure S4 shows total sleep time (Figures S4A and S4B), wake after sleep onset (Figures S4C and S4D), and slow wave sleep onset data during and after exposure (Figures S4E and S4F). Total sleep time and wake after sleep onset are reported as percent change from time-matched pre-exposure period for each hour, whereas slow wave sleep onset is reported as post-exposure values minus pre-exposure value. In male rats, smoke exposure significantly increased slow wave sleep onset (*p* < 0.05) relative to male rats exposed to filtered air during exposure, responses not evident in female rats. There were no significant effects of smoke exposure on total sleep time or wake after sleep onset in either male or female rats and no effect of smoke exposure on slow wave sleep onset after exposure in male rats.

**Fig. 5:**
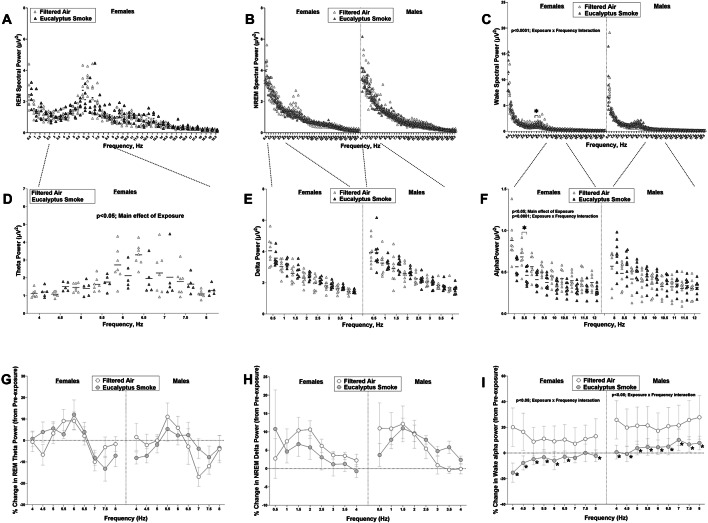
Eucalyptus smoke exposure significantly alters theta and alpha power. Electroencephalogram (EEG) data were recorded using implantable telemetry in male and female Sprague Dawley rats to measure polysomnography. Normalized discrete fast Fourier transform was used to evaluate spectral power for rapid-eye movement (REM) sleep, non-rapid-eye movement (NREM) sleep, and wake prior to (8 h; 11:00 AM to 7:00 PM), during (1 h; 9:30 to 10:30 AM), and after (8 h; 11:00 AM to 7:00 PM) filtered air or eucalyptus smoke exposure. Data were separated by sex. The following measurements were collected: (**a**) female REM spectral power during exposure (Note: not enough REM data in males to allow for REM spectral analysis) (**b**) NREM spectral power during exposure (**c**) Wake spectral power during exposure (**d**) female theta power, 4–8 Hz during exposure (**e**) delta power, 0.5–4 Hz during exposure (**f**) alpha power, 8–12 Hz during exposure (**g**) percent change post-exposure (relative to pre-exposure) in REM theta power (**h**) percent change post-exposure (relative to pre-exposure) in NREM delta power (**i**) percent change post-exposure (relative to pre-exposure) in wake alpha power. Data are mean ± SEM and analyzed by two-way repeated measures ANOVA with Šídák’s multiple comparisons test. Data during exposure are reported are reported for each frequency ; percent change data after exposure are also reported for each frequency . *p < 0.05, **p < 0.01, ****p < 0.0001. *n* = 8 per group

**Fig. 6:**
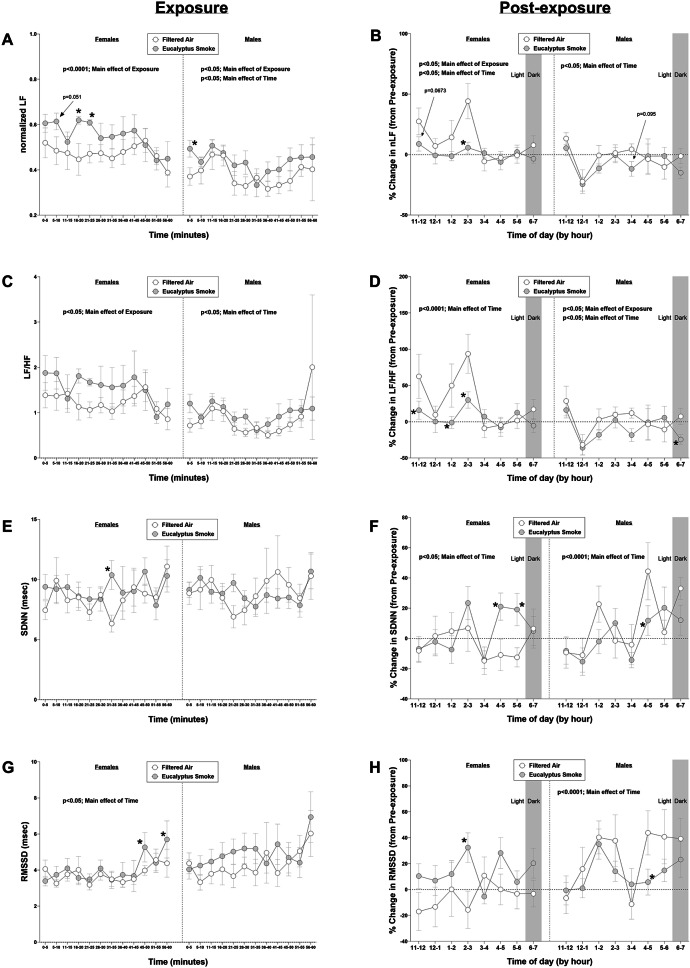
Eucalyptus smoke alters heart rate variability (HRV) parameters during and after exposure. Cardiovascular physiology was recorded using implantable telemetry in male and female Sprague Dawley rats to measure HRV prior to (8 h; 11:00 AM to 7:00 PM), during (1 h; 9:30 to 10:30 AM), and after (8 h; 11:00 AM to 7:00 PM) filtered air or eucalyptus smoke exposure. Data were separated by sex. The following measurements were collected: (**a**) normalized low frequency (nLF) in 5-min intervals during exposure (**b**) nLF percent change post-exposure (relative to pre-exposure) (**c**) ratio of low frequency to high frequency (LF/HF) in 5-intervals during exposure (**d**) LF/HF percent change post-exposure (relative to pre-exposure) (**e**) standard deviation of normal-to-normal heartbeats (SDNN) in 5-min intervals during exposure (**f**) SDNN percent change post-exposure (relative to pre-exposure) (**g**) root mean square of successive differences (RMSSD) in 5-min intervals during exposure and (**h**) RMSSD percent change post-exposure (relative to pre-exposure). Data are mean ± SEM and and were analyzed by a generalized linear mixed model. Data during exposure are reported in 5-min intervals; percent change data after exposure are reported in 1-hr intervals. *p < 0.05. *n* = 8 per group

Figure 6 shows spectral data for REM (Fig. 5A), NREM (Fig. 5B) and Wake (Fig. 5C), as well as corresponding data for theta (Fig. 5D), delta (Fig. 5E), and alpha oscillations (Fig. 5F), respectively, during the exposure period where data from smoke-exposed rats are compared to data from filtered air exposed rats in both males and females. There was a main effect of exposure in REM theta power in females (*p* < 0.01; *F*_1, 14_ = 9.589). There were no significant differences in NREM delta power in smoke-exposed rats relative to filtered air groups in both male and female rats. Smoke exposure did cause a significant difference in wake power in female rats relative to female rats exposed to filtered air, noted by an interaction between exposure and frequency (*F*_38, 532_ = 7.152; *p* < 0.0001), responses not evident in male rats. For alpha oscillations (8–12 Hz), a main effect of exposure (*F*_1, 14_ = 9.589; *p* < 0.01) and an interaction between frequency and exposure (*F*_8, 112_ = 5.351; *p* < 0.0001) was observed for females exposed to eucalyptus smoke compared with females exposed to filtered air, with a significant decrease in the 8.5 Hz range (*p* < 0.05). Additionally, when a percent change calculation was made comparing pre- and post-exposure periods, a frequency x exposure interaction (*F*_8, 112_ = 2.898; *p* < 0.01) was noted in females and males exposed to smoke, relative to sex-matched groups exposed to filtered air across the wake alpha power spectrum, with significant differences at most frequencies in both sexes (Fig. 5I). No significant differences were evident in REM theta (Fig. 5G) and NREM delta (Fig. 5H) power after exposure, when calculating percent change from the pre-exposure period. Figure S5 compares the pre- and post-exposure data for each exposure group for each sex. For REM power and theta power, period x frequency interactions were observed for all groups except when assessing theta power for males exposed to filtered air (Figure S5A-D). When evaluating NREM spectral power, a period (i.e., pre- and post-exposure periods) x frequency interaction was observed for males exposed to smoke (*p* < 0.0001) with post-hoc differences showing an increase at 0.5, 1, 1.5, 2.5 and 3 Hz (*p* < 0.05, Figure S5F). When delta power was assessed, a period x frequency interaction was observed for filtered air-exposed males (*F*_7,49_ = 2.588; *p* < 0.05) and a main effect of period (*F*_1, 7_ = 7.308; *p* < 0.05), with an increase at 0.5 and 1 Hz post- exposure (*p* < 0.01 and *p* < 0.05, respectively). A main effect of period was noted for males exposed to smoke (*F*_1, 7_= 14.77; *p* < 0.01) with an increase at 0.5, 1, 1.5 and 2 Hz (*p* < 0.01, *p* < 0.001, *p* < 0.0001, and *p* < 0.05, respectively; Figure S5H). These data suggest that exposure stress may enhance the drive for NREM sleep post- exposure in males.

Figure S6 shows wake and alpha spectral power pre- and post-exposure. For wake power in males exposed to filtered air, there was a period x frequency interaction (*F*_38, 266_ = 1.484; *p* < 0.05), while in males exposed to smoke a main effect of period was observed (*F*_1, 7_ = 6.518; *p* < 0.05; Figure S6B). When assessing alpha power pre- and post-exposure, a period x frequency interaction was observed (*F*_8, 56_ = 2.958; *p* < 0.01) with lower alpha power in the 8 Hz range (*p* < 0.0001) for smoke-exposed females (Figure S6C), but there was no such effect in males.

Figures S7 shows activity data during exposure and the percent change after exposure (relative to pre-exposure). During exposure, there was a significant overall effect of time on activity in females (*p* < 0.0001), but no overall effects in males. With respect to 5-min data in males, smoke exposure caused a tendency towards a significant decrease in activity at minutes 11–15 of the exposure regimen (*p* = 0.069), but there were no effects on 5-min activity data in females. After exposure, there was a significant overall effect of time (*p* < 0.0001) and a significant interaction between exposure and time (*p* < 0.0001) on activity in males, but no overall effect in females. Figure S8 correlates activity with NREM duration data. Activity significantly negatively correlated with NREM duration in both female and male rats exposed to either filtered air or smoke both during exposure (Figures S8A, C, E and G, *p* < 0.001, *p* < 0.05, *p* < 0.0001) and before and after exposure (Figures S8B, D, F and H, *p* < 0.0001).

### Heart rate variability during and after exposure

To determine if the heart rate and blood pressure responses were accompanied by changes in autonomic tone, HRV was assessed using the blood pressure signal before, during, and after exposure and is shown in Figs. 6 and S9. During exposure, there was a significant overall effect of exposure on nLF (*p* < 0.0001), nHF (*p* < 0.0001), vLF (*p* < 0.05), LF (*p* < 0.0001), HF (*p* < 0.05), and LF/HF (*p* < 0.05) and a significant overall effect of time on RMSSD (*p* < 0.05) and HF (*p* < 0.05) in females. In males during exposure, there was a significant overall effect of exposure on nLF (*p* < 0.05), nHF (*p* < 0.05), vLF (*p* < 0.05), LF (*p* < 0.0001), and HF (*p* < 0.0001) and a significant overall effect of time on nLF (*p* < 0.05), nHF (*p* < 0.05), LF (*p* < 0.05) and LF/HF (*p* < 0.05). With respect to frequency domain parameters at 5-minute intervals during exposure, smoke significantly increased nLF (and decreased normalized HF) at minutes 5–10, 16–20 and 21–25 in female rats relative to female rats exposed to filtered air (*p* < 0.05; Fig. 6A). In male rats during exposure, smoke exposure significantly increased nLF (and decreased normalized HF) at minutes 0–5 (p < 0.05) relative to male rats exposed to filtered air. There were no statistically significant changes during specific 5-min intervals in LF/HF (Fig. 6C) with exposure in either sex, although LF/HF followed a similar pattern as nLF, with average values greater in the smoke-exposed groups of both sexes relative to corresponding filtered air-exposed groups throughout the entire exposure regimen. ) Smoke exposure also caused significant increases in vLF (Figure S9C) at minutes 5–10 in female rats relative to female rats exposed to filtered air (*p* < 0.05) and at minutes 16–20 in male rats relative to male rats exposed to filtered air (*p* < 0.05). Smoke exposure also caused significant increases in LF (Figure S9E) at minutes 5–10, 11–15, 26–30 and 41–45 in female rats relative to female rats exposed to filtered air (*p* < 0.05) and at minutes 46–50 in male rats relative to male rats exposed to filtered air. Smoke exposure also caused significant increases in HF (Figure S9G) at minutes 11–15 and 41–45 in female rats relative to female rats exposed to filtered air (*p* < 0.05) and at minutes 41–45, and 46–50 in male rats relative to male rats exposed to filtered air.

After exposure in female rats, there was a significant overall effect of exposure on nLF (*p* < 0.05) and a tendency towards a significant overall effect of exposure on LF/HF (*p* = 0.0724) and RMSSD (*p* = 0.0766). There was also a significant overall effect of time on nLF (*p* < 0.05), nHF (p *p* < 0.05), LF (*p* < 0.05), LF/HF (*p* < 0.0001), and SDNN (*p* < 0.05) and a tendency towards a significant interaction between exposure and time in RMSSD (*p* = 0.0653) in females. In males, there was a significant overall effect of exposure on LF/HF (*p* < 0.05) and a significant overall effect of time on nLF (*p* = 0.0001), nHF (*p* < 0.0001), LF (*p* < 0.05), LF/HF (*p* < 0.05), SDNN (*p* < 0.0001) and RMSSD (*p* < 0.0001). There was also a tendency towards a significant overall effect of time (*p* = 0.0750) and a tendency towards a significant interaction between exposure and time (*p* = 0.0771) on vLF in males. Smoke exposure decreased nLF and increased nHF (reported as % change relative to time-matched pre-exposure values) at hours 2–3 PM (Figs. 6B and S9B; *p* < 0.05) and caused a tendency towards decreased nLF at hours 11AM-12PM (Figs. 6B and S9B; *p* = 0.0673) relative to female rats exposed to filtered air, with no significant effect of exposure on post-exposure nLF or nHF values in males. Smoke exposure in female rats also caused a tendency towards a significant decrease in vLF at hour 6–7 PM relative to female rats exposed to filtered air (Figure S9D; p = 0.054) whereas smoke exposure in male rats caused a significant decrease in vLF at hour 1–2 PM and a significant increase in vLF at hour 5–6 PM relative to male rats exposed to filtered air (Figure S9D; *p* < 0.05). Smoke exposure in female rats also caused significant decreases in LF/HF at hours 11AM-12 PM, 1–2 PM, and 2–3 PM and in male rats at hours 6–7 PM relative to their respective sex-matched filtered air controls (Fig. 6D; *p* < 0.05). Smoke exposure in male rats also caused a significant decrease in LF at hour 11AM-12 PM post-exposure (Figure S9F; *p* < 0.05), and in female rats, caused a significant increase in HF at hours 2–3 PM (Figure S9H; *p* < 0.05) relative to their respective sex-matched filtered air controls (*p* < 0.05).

With respect to time domain parameters of HRV, smoke exposure increased SDNN (Fig. 6E) at minutes 31–35 during exposure and RMSSD at minutes 46–50 and 56–60 (Fig. 6G) during exposure relative to female rats exposed to filtered air (*p* < 0.05), whereas there were no significant effects on these parameters in male rats during exposure. After exposure, smoke increased SDNN at hours 4–5 PM and 5–6 PM (Fig. 6F; *p* < 0.05) and caused significant increases in RMSSD at hour 2–3 PM (Fig. 6H; *p* < 0.05) relative to female rats exposed to filtered air. Smoke in male rats significantly decreased SDNN (Fig. 6F; *p* < 0.05) and RMSSD (Fig. 6H; *p* < 0.05) at hour 4–5 PM relative to male rats exposed to filtered air after exposure.

### Correlations of Cardiovascular, sleep and HRV parameters

**Fig. 7:**
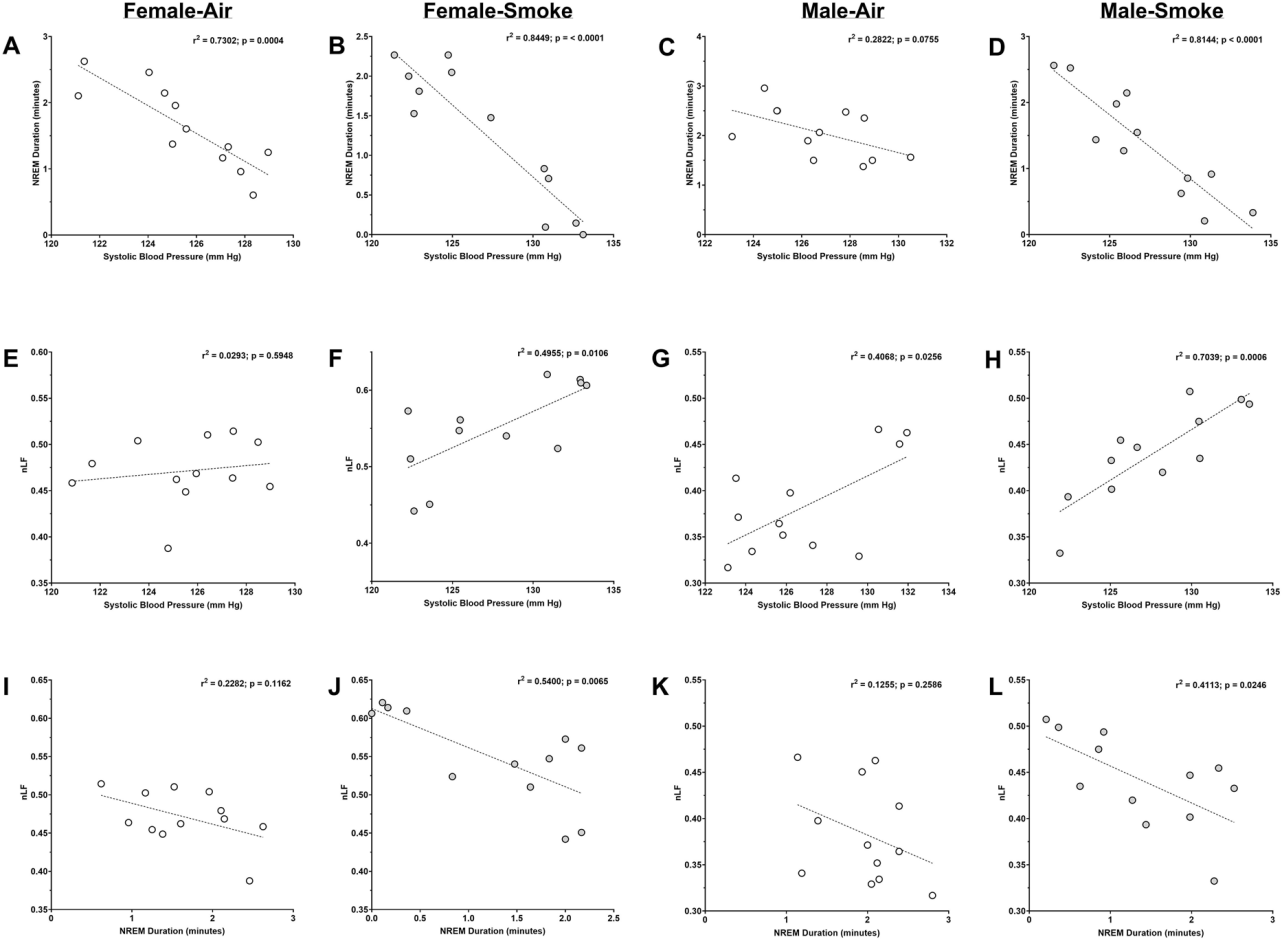
Eucalyptus smoke (ES) exposure elicits a strong negative correlation between NREM duration and systolic blood pressure, and NREM duration and nLF, and elicits a positive correlation between nLF and systolic blood pressure. The values for the variables NREM duration, systolic blood pressure, and nLF determined during exposure (9:30 AM to 10:30 AM) were correlated with one another. Data were separated by sex. The following correlations were evaluated: (**a**) NREM duration vs. systolic blood pressure, filtered air (FA) females (**b**) NREM duration vs. systolic blood pressure, eucalyptus smoke (ES) females (**c**) NREM duration vs. systolic blood pressure, FA males (**d**) NREM duration vs. systolic blood pressure, ES males (**e**) nLF vs. systolic blood pressure, FA females (**f**) nLF vs. systolic blood pressure, ES females (**g**) nLF vs. systolic blood pressure, FA males (**h**) nLF vs. systolic blood pressure, ES males (**i**) NREM duration vs. nLF, FA females (**j**) NREM duration vs. nLF, ES females (**k**) NREM duration vs. nLF, FA males, (**l**) NREM duration vs. nLF, ES males. Data were analyzed by simple linear regression. *n* = 8 per group. r^2^ values and significance levels are indicated

Fig. 7 and Tables 2, 3,4, and S1-S5 show correlation data for sleep vs. cardiovascular parameters, HRV vs. cardiovascular parameters, and HRV vs. sleep parameters. During exposure, NREM duration significantly negatively correlated with time-matched HR, SBP, DBP, and MAP in both filtered air and smoke-exposed female rats (*p* < 0.05), with correlations much stronger in smoke-exposed rats. Similar relationships were evident among male rats, with stronger correlations in smoke-exposed rats, although NREM duration did not significantly correlate with HR and MAP in male rats exposed to filtered air (Fig. 7; Table 2). Wake duration during exposure significantly positively correlated with time-matched HR, SBP, DBP, and MAP in both filtered air and smoke-exposed female rats (*p* < 0.05), with correlations again stronger in smoke-exposed rats. Similar relationships between wake duration and cardiovascular parameters were evident among male rats, with stronger correlations in smoke-exposed rats, although wake duration did not significantly correlate with HR, SBP and MAP in male rats exposed to filtered air (Fig. 7; Table 2). Both NREM and wake durations correlated in a similar fashion with PP in female rats exposed to either air or smoke (*p* < 0.05), with responses again stronger in smoke-exposed rats, but there were no significant correlations between either parameter and PP in either exposure group in male rats. The HRV marker reflecting sympathetic tone, i.e., nLF, significantly positively correlated with the cardiovascular parameters HR, SBP, DBP, and MAP in the smoke-exposed groups in both females and males (*p* < 0.05), but not in most corresponding air-exposed rats (Fig. 7; Table 3 and S1). nLF did significantly correlate with DBP and MAP in male rats exposed to filtered air, although correlations were again stronger in smoke-exposed male rats. nLF also significantly correlated with PP in female rats, exposed to smoke and had a tendency towards a significant correlation with PP in male rats exposed to smoke (*p* = 0.078)but there were no significant correlations between nLF and PP in the filtered air groups of either sex. LF/HF, another HRV marker of sympathetic tone, also significantly positively correlated with HR (*p* < 0.05) and had a tendency towards a significant correlation with SBP (*p* = 0.076) in smoke-exposed females, but not air exposed females (Table S1). LF/HF also significantly positively correlated with HR, SBP, DBP, and MAP and had a tendency towards a significant positive correlation in PP (*p* = 0.053) in smoke-exposed males. LF/HF also significantly correlated with SBP and DBP in air-exposed males, although the correlations were again stronger in smoke-exposed males. NREM duration significantly negatively correlated with time-matched nLF only in smoke-exposed female and male rats, but not corresponding air-exposed rats (*p* < 0.05; Fig. 7; Table 4 ). NREM duration also significantly negatively correlated with time-matched LF/HF only in female rats exposed to smoke (*p* < 0.05; Table S2) and had a tendency towards a significant negative correlation only in male rats exposed to smoke (*p* = 0.073). Wake duration significantly positively correlated with time-matched nLF in smoke-exposed female and male rats, but not corresponding air-exposed rats (*p* < 0.05; Table 4). Wake duration also significantly positively correlated with time-matched LF/HF only in smoke-exposed female rats (*p* < 0.05; Table S2) and had a tendency towards a significant positive correlation only in smoke-exposed male rats (*p* =0.066 ).

**Table 2:** Correlation analysis of sleep parameters vs. cardiovascular endpoints during exposure

	Female-Air	Female-Smoke	Male-Air	Male-Smoke
NREM Duration (min) vs. Cardiovascular Endpoints				
Heart Rate (bpm)	r^2^ = 0.6519; *p* = 0.0015*	r^2^ = 0.9118; *p* < 0.0001*	r^2^ = 0.2531; *p* = 0.0955	r^2^ = 0.8306; *p* < 0.0001*
Diastolic BP (mmHg)	r^2^ = 0.7202; *p* = 0.0005*	r^2^ = 0.8253; *p* < 0.0001*	r^2^ = 0.3731; *p* = 0.0349*	r^2^ = 0.8651; *p* < 0.0001*
Pulse Pressure (mmHg)	r^2=^0.6215; *p* = 0.0023*	r^2^ = 0.6671; *p* = 0.0012*	r^2^ = 0.0287; *p* = 0.5988	r^2^ = 0.1329; *p* = 0.2440
MAP (mmHg)	r^2^ = 0.7319; *p* = 0.0004*	r^2^ = 0.8453; *p* < 0.0001*	r^2^ = 0.3059; *p* = 0.0621	r^2^ = 0.8468; *p* < 0.0001*
Wake Duration (min) vs. Cardiovascular Endpoints				
Heart Rate (bpm)	r^2^ = 0.7101; *p* = 0.0006*	r^2^ = 0.9190; *p* < 0.0001*	r^2^ = 0.1650; *p* = 0.1901	r^2^ = 0.8734; *p* < 0.0001*
Systolic BP (mmHg)	r^2^ = 0.6672; *p* = 0.0012*	r^2^ = 0.8429; *p* < 0.0001*	r^2^ = 0.2967; *p* = 0.0671	r^2^ = 0.7931; *p* = 0.0001*
Diastolic BP (mmHg)	r^2^ = 0.6344; *p* = 0.0019*	r^2^ = 0.7874; *p* = 0.0001*	r^2^ = 0.3356; *p* = 0.0484*	r^2^ = 0.8871; *p* < 0.0001*
Pulse Pressure (mmHg)	r^2^ = 0.6045; *p* = 0.0029*	r^2^ = 0.7222; *p* = 0.0005*	r^2^ = 0.00003; *p* = 0.9956	r^2^ = 0.0701; *p* = 0.4055
MAP (mmHg)	r^2^ = 0.6548; *p* = 0.0014*	r^2^ = 0.8304; *p* < 0.0001*	r^2^ = 0.2837; *p* = 0.0746	r^2^ = 0.8444; *p* < 0.0001*

Correlations were derived from plots of five-minute group averages of NREM duration (min) or Wake duration (min) vs. group averages of concurrent measures of cardiovascular physiology per 5-minute period over the course of the 1-hr exposure period (12 5-min averages per group)

**Table 3:** Correlation analysis of a frequency domain measure of HRV vs. cardiovascular endpoints during exposure

nLF vs. Cardiovascular Endpoints				
	Female-Air	Female-Smoke	Male-Air	Male-Smoke
Heart Rate (bpm)	r^2^ = 0.0041; *p* = 0.8435	r^2^ = 0.6560; *p* = 0.0014*	r^2^ = 0.2165; *p* = 0.1274	r^2^ = 0.6494; *p* = 0.0016*
Diastolic BP (mmHg)	r^2^ = 0.0332; *p* = 0.5710	r^2^ = 0.3943; *p* = 0.0288*	r^2^ = 0.4360; *p* = 0.0194*	r^2^ = 0.6343; *p* = 0.0019*
Pulse Pressure (mmHg)	r^2^ = 0.0186; *p* = 0.6726	r^2^ = 0.4980; *p* = 0.0103*	r^2^ = 0.0805; *p* = 0.3715	r^2^ = 0.2778; *p* = 0.0783
MAP (mmHg)	r^2^ = 0.0290; *p* = 0.5967	r^2^ = 0.4324; *p* = 0.0201*	r^2^ = 0.3717; *p* = 0.0353*	r^2^ = 0.6739; *p* = 0.0011*

Correlations were derived from plots of five-minute averages of nLF vs. concurrent measures of cardiovascular physiology per 5-minute period for each animal over the course of 1-hr of exposure (12 values per animal)

**Table 4:** Correlation analysis of sleep parameter vs. frequency domain measure of HRV during exposure

Wake Duration vs. nLF				
	Female-Air	Female-Smoke	Male-Air	Male-Smoke
nLF	r^2^ = 0.1589; *p* = 0.1993	r^2^ = 0.5967; *p* = 0.0032*	r^2^ = 0.0815; *p* = 0.3686	r^2^ = 0.4200; *p* = 0.0227*

Correlations were derived from plots of five-minute averages of wake duration vs. concurrent measures of nLF per 5-minute period for each animal over the course of 1-hr of exposure (12 values per animal)

After exposure, NREM duration (normalized to pre-exposure) also significantly negatively correlated with time-matched HR, SBP, DBP, and MAP in female air-exposed rats, and both male exposure groups (*p* < 0.05), with correlations stronger in male smoke-exposed rats (Table S3). In the female-smoke group, NREM was significantly negatively correlated only with HR and not any other cardiovascular parameter, although it had a tendency towards a significant negative correlation with DBP (*p* = 0.063). Likewise, wake duration post exposure significantly negatively correlated with time-matched HR, SBP, DBP, and MAP in female air-exposed rats, and both male exposure groups (*p* < 0.05), with correlations stronger in male smoke-exposed rats (Table S3). Wake duration in the female-smoke group also significantly negatively correlated only with HR and not any other cardiovascular parameter. There were no significant correlations in either NREM duration or wake duration with PP in either males or females of both exposure groups. nLF significantly positively correlated with most cardiovascular parameters including HR, SBP, DBP, and MAP in the smoke-exposed groups in both males and females (*p* < 0.05), but not in the corresponding air-exposed rats, although the relationship between nLF and SBP in the female-smoke group only had a tendency towards a significant correlation (*p* = 0.073; Table S4). LF/HF in both the female and male smoke-exposed groups significantly positively correlated only with HR and not any other cardiovascular parameter (*p* < 0.05; Table S4), although there was a tendency towards a significant positive correlation between LF/HF and DBP in the female-air group (*p* = 0.0576), and LF/HF and SBP (*p* = 0.0573), DBP (*p* = 0.0587), and MAP (0.0516) in the male-smoke group (Table S4). There were no significant correlations between nLF or LF/HF with PP in any exposure group. NREM duration post exposure significantly negatively correlated with time-matched nLF and LF/HF only in the smoke-exposed female and male rats, but not corresponding air-exposed rats (*p* < 0.05; Table S5). Likewise, wake duration significantly positively correlated with time-matched nLF and LF/HF in smoke-exposed female and male rats, and in the air-exposed male rats (*p* < 0.05), although the relationship was stronger in smoke-exposed male rats relative to air-exposed male rats (Table S5).

### Blood pressure variability during and after exposure

**Fig. 8:**
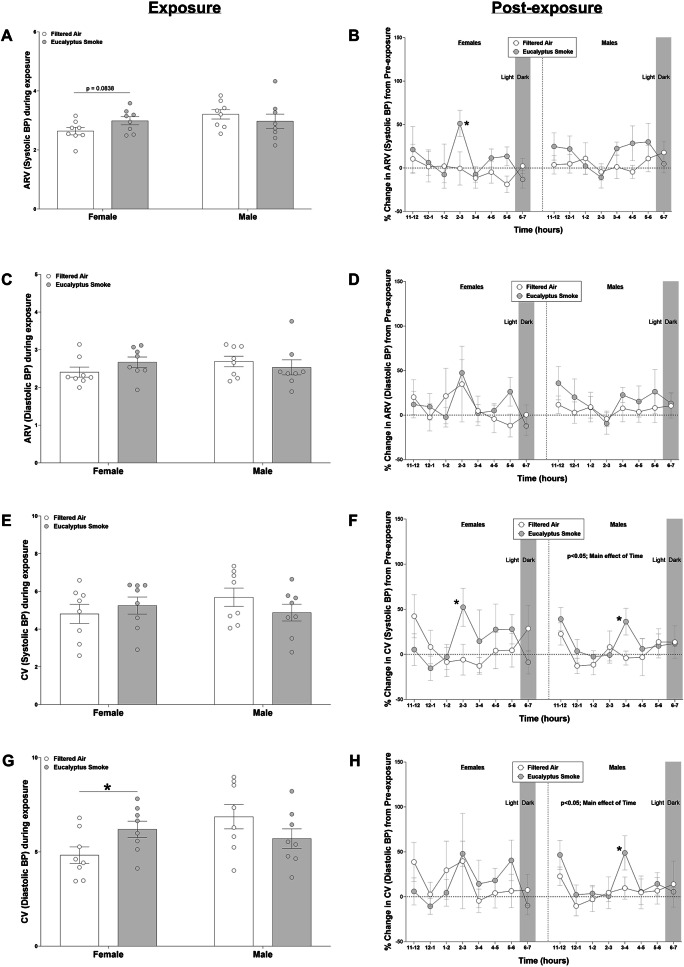
Eucalyptus smoke elicits higher coefficient of variation for diastolic blood pressure in females during exposure. Cardiovascular physiology was recorded using implantable telemetry in male and female Sprague Dawley rats to measure blood pressure variability prior to (8 h; 11:00 AM to 7:00 PM), during (1 h; 9:30 to 10:30 AM), and after (8 h; 11:00 AM to 7:00 PM) filtered air or eucalyptus smoke exposure. Data were separated by sex. The following measurements were calculated: average real variability (ARV) of systolic blood pressure during exposure (**a**) percent change of ARV of systolic blood pressure post-exposure (relative to pre-exposure) (**b**) ARV of diastolic blood pressure during exposure (**c**) percent change of ARV of diastolic blood pressure post-exposure (relative to pre-exposure) (**d**) coefficient of variation (CV) for systolic blood pressure (**e**) percent change of CV of systolic blood pressure post-exposure (relative to pre-exposure) (**f**) CV for diastolic blood pressure (**g**) percent change of CV for diastolic blood pressure post-exposure (relative to pre-exposure) (**h**). Data are mean ± SEM and analyzed by Student’s t-test (figures a, c, e. and g), or by a generalized linear mixed model (figures b, d, f, and h). Data during exposure are reported as averages during the entire exposure period; percent change data after exposure are reported in 1-hr intervals. *p < 0.05. *n* = 8 per group

Blood pressure variability data are shown in Figs. 8 and S10. During exposure, female rats exposed to eucalyptus smoke had significant increases in the CV (*p* < 0.05; Fig. 8G) and SD (*p* < 0.05; Figure S10C) of diastolic blood pressure and a tendency towards an increase in the ARV of systolic blood pressure (*p* = 0.0838; Fig. 8A) relative to female rats exposed to filtered air., with no significant changes in males rats in either exposure group during exposure. After exposure, in males, there was a significant overall effect of time on the CV of systolic (*p* < 0.05) and diastolic (*p* < 0.05) blood pressure and SD of systolic blood pressure (*p* < 0.05). There were no significant overall effects or interactions in any BPV parameter in females. On an hourly basis post-exposure (reported as % change from time-matched pre-exposure period for each hour), smoke exposure significantly increased the ARV (Fig. 8B; *p* < 0.05) and CV (Fig. 8F; *p* < 0.05) of systolic blood pressure at hour 2–3 PM in female rats, whereas smoke exposure significantly increased the CV and SD of systolic and diastolic blood pressure at hour 3–4 PM in male rats (Fig. 8F and H and Figures S10B and S10D; *p* < 0.05) relative to their respective sex-matched filtered air controls.

### Lung and systemic factors one day after exposure

Smoke exposure in female rats caused a significant increase in lung neutrophils (*p* < 0.05) and significant decreases in lung GGT and NAG (*p* < 0.05) relative to female rats exposed to filtered air, responses not evident in male rats (Table S6). Smoke exposed females also had significant increases in whole blood WBCs (*p* < 0.05) and a tendency towards a significant increase in whole blood lymphocytes (*p* = 0.0563) relative to female rats exposed to filtered air, whereas smoke exposure in male rats caused significant increases in whole blood MCH and MCHC (*p* < 0.05) and a significant decrease in whole blood RBCs (*p* < 0.05) relative to male rats exposed to filtered air. Smoke exposure in female rats also caused significant increases in serum ALP and IL-10 (*p* < 0.05) and a tendency towards a significant increase in TNF-α (*p* < 0.0501) relative to female rats exposed to filtered air, whereas smoke-exposed male rats had significant increases in CRP, IFN-γ, IL-4, and IL-10 (*p* < 0.05) relative to male rats exposed to filtered air.

### Gene expression in the hypothalamus one day after exposure

There were no significant changes in gene expression in both sexes (Tables S7 and S8) in response to eucalyptus smoke exposure, except for a decrease in *Egr3* in males (*p* < 0.05; Table S8).

## Discussion

This study examined the impacts of a single inhalation exposure to eucalyptus smoke, a model wildfire combustion emission, on cardiovascular responses, particularly blood pressure, and whether such responses were related to alterations in sleep-wake behavior and architecture. In addition to an overall effect on HR and PP in females and PP in males, exposure to eucalyptus smoke increased heart rate and systolic blood pressure in both female and male rats, and diastolic blood pressure in male rats, at the beginning of the exposure regimen. By the middle of the exposure regimen, however, smoke had induced a decrease in HR, and SBP and DBP in male rats. These findings are consistent with our previous findings with other sources of air pollution including peat smoke [57], ozone [64], and diesel exhaust [65] that were characterized by immediate short-term increases at the beginning of the exposure regimen followed by rebound and compensatory decreases in responses during or shortly after exposure. Although the increases were transient, these findings point to a potential for cardiovascular effects with acute exposure in people. Hypertension has previously been associated with exposure to smoke from forest fires in Athens, Greece in 2021 [68], peat fires in North Carolina, USA in 2011 [69], and agricultural fires in India from 2015 to 2016 [70], while exposure to wildfire PM in Australia from 2000 to 2008 was associated with differential methylation of genes related to blood pressure [71]. Exposure to smoke from a related source, i.e., biomass burning used for cookstoves or indoor heating, has also been linked to hypertension [72, 73]. However, other epidemiological [74] and controlled human exposure [75] studies of the health effects of wildfire-related smoke reported various adverse cardiovascular effects but no significant changes in blood pressure while blood pressure data among wildfire-fighting firefighters are mixed [76–78] and difficult to disentangle from other occupation-related factors (e.g., stress). Nonetheless, a recent metanalysis of global findings established strong linkages between hypertension and exposure to multiple ambient air pollutants including PM_2.5_, SO_2_, and NO_2_ [35], all major constituents of wildfire smoke, and we have previously demonstrated blood pressure increases in rats with exposure to peat [57] and eucalyptus smoke [56, 79]. The precise reasons for the divergence in diastolic blood pressure responses to eucalyptus smoke among sexes in the present study are unclear. Studies have shown that changes in systolic and diastolic blood pressure in response to air pollution exposure are not necessarily aligned [80] and recent studies point to larger changes in blood pressure in response to air pollution in males compared to females [35, 81]. Moreover, a recent study determined that diastolic blood pressure was more sensitive to ambient PM_2.5_ exposure than systolic blood pressure in a mixed sex cohort [82]. By contrast, female rats exposed to eucalyptus smoke in the present study, but not male rats, had exposure period increases in pulse pressure, which, arithmetically, is the difference between systolic and diastolic blood pressure, and an indirect measure of arterial stiffness and predictor of cardiovascular risk [83]. This finding is consistent with a recent study that demonstrated increases in pulse pressure in women who were exposed to biomass combustion emissions indoors and who had greater increases in systolic compared to diastolic blood pressure [84]. Interestingly, eucalyptus smoke-exposed female rats, but not male rats, in the present study, also had increases in blood pressure variability, a phenomenon also linked with increased arterial stiffness and elevated cardiovascular risk [85]. Higher blood pressure variability and greater pulse pressure may be a manifestation of baroreflex dysfunction [86], which was not measured in the present study. Nonetheless, these findings are consistent with a study that showed that woodsmoke exposure in a small mixed-sex cohort increased arterial stiffness [75] which was measured using pulsed wave analysis of the right radial artery.

There was an overall effect of smoke exposure on SBP, DBP and MAP in both male and female rats after exposure. Male rats exposed to eucalyptus smoke also had hourly decreases in heart rate and systolic and diastolic blood pressure relative to filtered air controls, with more muted responses among females. Such decreases are consistent with recent findings in people, including one study which found decreases in systolic blood pressure in healthy male and female adults 30 min after a 2-hour exposure to 3-stone fire emissions [87] and a second study which found decreases in heart rate and systolic and diastolic blood pressure in mostly male wildland firefighters the morning after a work shift fighting fires [88]. Thus, although blood pressure responses to an acute exposure to wildfire-related smoke are transient, nuanced and likely influenced by fuel type, pre-existing disease, sex, and other factors, evidence from the present and prior studies suggests that exposure increases risk for hypertension.

Animals exposed to filtered air in the present study began to sleep almost immediately after placement into the exposure chamber, whereas exposure to eucalyptus smoke disrupted this pattern, especially during the first part of the exposure regimen, decreasing NREM sleep and increasing wake duration in male and to a lesser extent female rats. In addition to an overall effect on REM duration in both males and females, smoke exposure reduced total REM time in males and REM theta power and wake alpha power in females for the entire exposure period, while also reducing average REM duration for two five-minute periods in both male and female rats during the latter part of the exposure regimen. Eucalyptus smoke exposure also delayed the onset of NREM sleep in males, which corresponds with the increase in wake duration observed during exposure. These findings are consistent with multiple studies that have linked exposure to individual air pollutants including ultrafine, fine and coarse PM, the gases O_3_, NO_2_, and SO_2_ [89–95], as well as emissions from woodburning stoves [96, 97] to sleep disruption (e.g., reduced sleep duration, fragmented sleep) in people. Similar findings were reported with ozone exposure in rats [51, 52, 98]. NREM sleep, which is characterized by high amplitude, low frequency EEG activity, minimal muscle movement, and reduced heart rate, blood pressure and sympathetic nervous system tone [99], makes up about 80% of the normal sleep cycle, and is necessary for declarative and motor memory consolidation and body rejuvenation [100]. REM sleep, which is characterized by high frequency, low amplitude EEG activity with frequent theta oscillations, dreaming, muscle atonia and fluctuating heart rate, blood pressure and sympathetic nervous system tone [99], accounts for the remainder of the sleep cycle, and is important for procedural, implicit, and spatial learning and memory, as well as emotional well-being and regulation [101, 102]. Reduced NREM sleep duration and disrupted NREM delta wave sleep have been linked with development of hypertension [103, 104] and increased risk of coronary heart disease and cardiovascular mortality, respectively [105], while perturbations in REM sleep have been linked with increased risk of atrial fibrillation [106, 107] and all-cause mortality [108]. The increased risk for hypertension is likely related to the fact that NREM sleep is necessary for sympathetic activity to decrease and for the concomitant drops in heart rate and blood pressure associated with normal nocturnal blood pressure dipping [109]. Disruptions in REM sleep are also likely to promote hypertension because, although sympathetic activity and blood pressure increase during this period, baroreflex control is much more effective at stemming blood pressure increases during REM compared to other periods [109]. Repeated disruption in REM and NREM sleep over time with long-term exposure to air pollution may elicit other serious adverse cardiovascular effects as well. For example, deliberate sleep fragmentation has been shown to cause endothelial dysfunction, arterial stiffness, and vascular remodeling in wild type mice [110], and accelerated transition to heart failure in mice with hypertrophic cardiomyopathy [111]. After the animals were returned to their home cages following exposure, both male and female smoke-exposed rats had a decrease in wake alpha power during the entire post-exposure monitoring period demonstrating a diminished drive for wakefulness, potentially in response to mild sleep deprivation during exposure and a transition to NREM sleep in an acute setting [112]. Reductions in alpha power have recently been documented in thalamic stroke patients who also had long sleep duration and concomitant increases in parasympathetic tone [113], consistent with the post-exposure findings in the present study. After exposure, there was also an overall effect of exposure on NREM duration in females and males had increases in REM bout duration. These post-exposure increases are suggestive of a rebound response to overcome the sleep deficit endured during exposure, a phenomenon previously linked with other sleep disruptors including indoor light in people [114] and noise in rats [115]. The concomitant drops in blood pressure and heart rate after exposure in males further support an increased proclivity for sleep after smoke exposure. Differences in sleep responses among males and females, like those reported in the present study, are not unusual and consistent with variable sleep responses to air pollution in men and women reported in recent studies [116, 117]. 

There was an overall effect of exposure on the HRV markers nLF and LF/HF and significant increases in nLF in the early part of the exposure regimen, suggestive of increased sympathetic nervous system tone in both female and male rats, although responses were more prominent in female rats. While there remains some controversy regarding the validity of their linkage with sympathetic tone [118], particularly LF which has been linked to baroreceptor activity [119], nLF and LF/HF are generally taken as measures of sympathetic activity [120] and sympathovagal balance [121], respectively. nHF, which reflects parasympathetic tone, and is arithmetically the value of nLF subtracted from 1 [121], decreased with smoke exposure, also indicating increased sympathetic tone. Importantly, heart rate and blood pressure responses to eucalyptus smoke, but not filtered air, were significantly positively correlated with nLF and LF/HF in both male and female rats suggesting potential mediation, in part, by the sympathetic nervous system. Exposure to eucalyptus smoke also caused increases in vLF, a similarly controversial marker, during the early part of the exposure regimen in both female and male rats, although recent studies have linked it with parasympathetic tone [122]. These findings are consistent with a previous study that demonstrated decreases in HRV, reflecting increased sympathetic tone, in a small group of people (8 males and 6 females) immediately after a 3-hour exposure to wood smoke [75]. The sympathetic nervous system influences heart rate by increasing the firing rate of the sinus node [123] and elevates blood pressure by increasing contractility of the cardiac muscle and by promoting vasoconstriction [124]. Elevated cardiac sympathetic tone, as indicated by low HRV, increases lifetime cardiovascular risk [125]. These findings suggest that exposure to eucalyptus smoke caused a transient window of elevated cardiovascular risk in both sexes of otherwise young, healthy rats. There was also an overall effect of smoke exposure on nLF in female rats and LF/HF in male rats after exposure. Furthermore, heart rate and blood pressure significantly correlated with nLF and heart rate significantly correlated with LF/HF in both smoke-exposed male and female rats after exposure, suggesting that the functional responses after exposure were related to the relative level of sympathetic tone, paralleling the relationships during exposure. Such post-exposure effects may reflect vagally mediated rebound decreases following the short-term increases in sympathetic tone during exposure as suggested by the early hourly post-exposure increases in parasympathetic tone evident in female rates in particular (i.e., decreased nLF and LF/HF and increased nHF and RMSSD), similar to responses to other stressors including physical exercise [126] and psychological stress [127].

Although there were some differences among sexes in the present study, the cardiovascular, sleep, and sympathetic parameters largely became much more significantly correlated with one another in the smoke exposure group relative to the filtered air group both during and after exposure suggesting that the cardiovascular responses caused by exposure to smoke may have been driven in part by circadian disruption and associated changes in sympathetic nervous system tone. Importantly, these subtle relationships may be more sensitive markers of air pollution health effects than frank changes in function. Sleep disorders such as obstructive sleep apnea increase sympathetic tone, disturbing the normal circadian pattern of autonomic nervous system function characterized by higher relative parasympathetic tone during the nighttime sleep period [128]. Nightshift work [129], extended work shifts [130], simulated nightshift work [131] and exposure to artificial light during nighttime [132], all associated with poor sleep quality, similarly increase relative cardiac sympathetic tone. It was recently determined that air pollution exposure-induced disruptions in sleep are also accompanied by increases in cardiac sympathetic tone [133]. An increase in cardiac sympathetic tone secondary to sleep disruption may trigger or worsen cardiovascular disease. For example, individuals with loss of diurnal rhythm in autonomic tone and high sympathetic tone throughout the day are at increased risk for atherosclerotic cardiovascular disease [134]. In mice, disruption of the circadian clock by gene targeting of the positive regulators of circadian rhythm Bmal1 and Clock accelerates atherosclerosis progression [135] and cardiomyopathy [136], respectively. Sleep-disruption triggered cardiovascular responses may involve activation of a circadian-sympathetic-heart axis involving modulation of the sympathetic control center of the brain in the hypothalamus [137]. In the present study, there were no changes in circadian rhythm gene expression in the hypothalamus of females measured one day after exposure, although smoke exposure did decrease *Egr3* expression in males. Recently, blood levels of *Egr3*, which serves as a transcriptional regulator of genes in the suprachiasmatic nucleus (SCN), the main pacemaker of the brain that also regulates the sleep-wake cycle in response to light input from the retina [138, 139], were found to be downregulated in nightshift working nurses compared to daytime workers and negatively correlated with cortisol, a key stress hormone [141]. The mechanisms by which air pollution inhalation may trigger disruption in sleep and circadian rhythm, however, are unclear. Interestingly, exposure of mice to formalin vapor, which contains formaldehyde, a component of wildfire [141] and eucalyptus [142] smoke, caused arousal from sleep, responses that were absent in mice lacking the gene for the nociceptive cation channel transient receptor potential ankyrin 1 (*Trpa1*; 143, 144). TRPA1 mediates airway responses to irritants like formaldehyde [145] and, as we and others have demonstrated, the cardiovascular and sympathetic responses to inhaled air pollutants [146–151]. Airway TRPA1 was likely activated during exposures in the present study as eucalyptus smoke contains multiple other TRPA1 agonists including acrolein, acetaldehyde, and crotonaldehyde [142, 152, 153], although studies that target TRPA1 would be needed to confirm this phenomenon and its relationship with sleep and cardiovascular function.

While the immediacy of the changes along with the HRV findings suggest that increased sympathetic tone is the likeliest explanation for the cardiovascular responses during exposure, these findings do not preclude a role for inflammation and/or injury, which although not measured immediately after exposure in the present study, were evident to some degree in the lung and systemic circulation of both male and female smoke-exposed rats one day after exposure. The responses in females were particularly acute and included increases in lung neutrophils, circulating lymphocytes and pro-inflammatory cytokines indicating that even a single exposure to eucalyptus smoke has the capacity to elicit systemic pro-inflammatory responses in both males and females. Interestingly, one recent study found that air pollution exposure-induced sleep disturbances were linked with inflammation in a small cohort consisting mostly of women [133]. As such, repeated inflammatory responses resulting from episodic exposure to air pollution may contribute to impaired sleep over time and increased risk for cardiovascular disease. Exposure to smoke also caused a decrease in core body temperature only in females at the beginning of the exposure period. The impacts of this change are unclear, although separate exposures to ozone and particulate matter have previously been linked to hypothermic responses in rats that had coincident cardiovascular responses [154].

Although not directly compared statistically, there were some qualitative differences in responses to eucalyptus smoke across sex (summarized in Table 5), the exact reasons for which are unclear and require further study. While innate physical differences (e.g., lung volume and deposition, body weight) are partly responsible for the divergence in responsiveness among sexes, biological differences (e.g., endocrine/hormonal) undoubtedly also play a role [155]. Previous studies have indicated that the female hormone estrogen modifies lung responses to ozone air pollution in mice [156] and has mixed effects on sleep in people with some studies demonstrating a positive association with sleep efficiency and decreased awakening [157]. Estrogen and the male androgenic hormone testosterone have various sometimes divergent effects on cardiomyocyte and vascular function that vary by life stage [158, 159]. Testosterone has also been linked with altered circadian rhythm activity in mice, responses that also varied in young and old male mice [160]. Furthermore, estrogen and testosterone have differential impacts on the hypothalamic pituitary axis, which plays a substantial role in the cardiovascular effects of air pollution [161]. An added layer of complexity is estrous cyclicity, which was not assessed in the present study, and has been shown previously to modify responses to air pollution in female mice [162], and circadian rhythm [163], and cardiac autonomic tone in women [164]. Finally, the more pronounced smoke-induced temperature response during exposure and inflammatory response one day after exposure in females suggest that the mechanisms that drove the cardiovascular responses in females may be more complex and multi-factorial.

**Table 5:** Summary of the cardiovascular, sleep, and autonomic responses and their relationships during and after exposure to eucalyptus smoke relative to filtered air in female and male rats

	Female	Male
Cardiovascular Function	*During Exposure* • Overall effect on HR, PP • Early ↑ HR, SBP • ↑PP and BPV throughout *After Exposure* • Overall effect on SBP, DBP, MAP • Hour-by-hour ↑ and ↓BPV	*During Exposure* • Overall effect on PP • ↑ and then ↓ in HR, SBP, DBP and MAP *After Exposure* • Overall effect on SBP, DBP, MAP • Hour-by-hour ↓ SBP, DBP, PP, MAP • Hour-by-hour ↑BPV
Sleep	*During Exposure* • Overall effect on REM duration • Late ↓ in REM duration • Altered REM Theta Power, Wake Power and Wake Alpha Power *After Exposure* • Overall effect on NREM duration • ↓ REM duration in early hrs • ↑ NREM duration in later hr • ↓ in Wake Alpha Power	*During Exposure* • Overall effect on REM duration • Early ↓ in NREM duration, late ↓ in REM, ↓ total REM time • Early ↑ in Wake duration • ↑ in SWS Onset *After Exposure* • ↓ REM duration in early hrs • ↑ REM bout duration overall • ↓ in Wake Alpha Power
Autonomic Tone	*During Exposure* • Overall effect on nLF, LF/HF, nHF, vLF, LF, and HF • Early ↑ nLF, vLF, ↓ nHF • Periodic ↑ LF, HF, SDNN, RMSSD *After Exposure* • Overall effect on nLF • Hour-by-hour ↓ nLF, LF/HF, ↑ nHF, HF, SDNN, RMSSD	*During Exposure* • Overall effect on nLF, nHF, vLF, LF, and HF • Early ↑ nLF, vLF, ↓ nHF • Late ↑ LF, HF *After Exposure* • Overall effect on LF/HF • Hour-by-hour ↓ and ↑ vLF, ↓ SDNN, RMSSD, LF, LF/HF
Sleep vs. Cardiovascular	*During Exposure* • NREM, Wake Duration more strongly correlated w/ HR, SBP, DBP, PP, MAP *After Exposure* • NREM, Wake Duration less strongly correlated w/ HR, SBP, DBP, PP, MAP	*During Exposure* • NREM, Wake Duration more strongly correlated w/ HR, SBP, DBP, MAP *After Exposure* • NREM, Wake Duration more strongly correlated w/ HR, SBP, DBP, MAP
Autonomics vs. Cardiovascular	*During Exposure* • nLF more strongly correlated w/ HR, SBP, DBP, PP, MAP • LF/HF strongly correlated w/ HR *After Exposure* • nLF more strongly correlated w/ HR, DBP, MAP • LF/HF more strongly correlated w/ HR	*During Exposure* • nLF, LF/HF more strongly correlated with HR, SBP, DBP, MAP *After Exposure* • nLF more strongly correlated w/ HR, SBP, DBP, MAP • LF/HF more strongly correlated w/ HR
Sleep vs. Autonomics	*During Exposure* • NREM, Wake duration more strongly correlated with nLF, LF/HF *After Exposure* • NREM, Wake duration more strongly correlated w/ nLF, LF/HF	*During Exposure* • NREM, Wake duration strongly correlated with nLF *After Exposure* • NREM, Wake duration more strongly correlated w/ nLF, LF/HF

*HR* heart rate, *PP* pulse pressure, *SBP* systolic blood pressure, blood pressure variability, *DBP* diastolic blood pressure, *MAP* mean arterial pressure, *REM* rapid eye movement sleep, *NREM* non-rapid eye movement sleep, *LF* low frequency domain of heart rate variability, *HF* high frequency domain of heart rate variability, *LF/HF* ratio of LF to HF, *nLF* normalized LF, *nHF* normalized HF, *vLF* very low frequency LF, *SDNN* standard deviation of the RR interval, *RMSSD* square root of the mean of squared differences of adjacent RR intervals

The present study was limited by several factors. Although rodents have periods which can be considered “active” and “resting,” their sleep cycle is polyphasic [165], whereas humans sleep nearly continuously throughout the night and are awake during daytime [166].The electromyogram, a measure of muscle activity used to confirm wakefulness in sleep EEG studies, was not measured in the present study due in part to the type of implant used, which has only two biopotential leads, both of which were used for recording the EEG. However, locomotor activity, an inexact surrogate metric for wakefulness also recorded by the telemeter, significantly correlated with sleep metrics including NREM and wake duration. In addition, during the scoring process, power band visualizations were used to confirm vigilance state parameters as a second measure to confirm the differences between NREM, REM and wakefulness. Blood pressure and heart rate responses, also recorded by the dual implant, have distinct diurnal patterns that align with sleep and wakefulness in people and in the present study strongly correlated with sleep metrics irrespective of exposure group and sex. The present study also relied on analysis of peaks in the blood pressure waveform to determine HRV, an assessment that produces findings that may not always agree with HRV data from RR analysis of the ECG [167]. However, we previously demonstrated changes in HRV markers indicating increased sympathetic tone in rats during exposure to the air pollutant acrolein, a component of wildfire smoke; these effects were determined by analyzing the RR interval of the ECG [168]. The experimental animals were exposed during the first part of the 12-hour light cycle as takes place with most rodent studies, which disrupted their normal sleep period, and as such does not fully mirror day-time exposure in awake humans, although appreciable nighttime intrusion of PM into homes takes place during active fires. In addition, although acquired continuously during exposure, sleep data were only recorded for 4 min every 10 min before and after exposure, which may have limited the robustness of the sleep determinations during those periods, particularly when assessing sleep-wake architecture. Furthermore, the assessment of sleep data was limited to the exposure period and the immediate sleep periods before and after one exposure, and thus the impacts of exposure on subsequent sleep periods days after exposure is unclear. The present study also did not assess estrous cyclicity in the females, which has previously been shown to influence both sleep [169] and cardiovascular function [170] in rats. Moreover, assessments of circadian genes in hypothalamic tissue and markers of injury and inflammation one day after exposure missed likely responses shortly after exposure and were limited by the smaller group size in the un-telemetered female smoke-exposed rats. Finally, despite efforts to ensure the same exposure concentration, the average smoke PM_2.5_ concentration in the female exposures was over 250 µg/m^3^ higher than the average concentration during the male exposures. The extent to which this difference in exposure level accounted for the variability in one or more endpoints across sex is unclear.

## Conclusions

In conclusion, findings from the present study provide previously undescribed evidence that suggests that sleep disruption and associated sympathetic effects may have contributed to the observed hypertensive responses to inhaled air pollution given their concurrence and established pathophysiologic linkages. A role for sleep is further bolstered by the strong correlation among cardiovascular, sleep, and sympathetic responses both during and after exposure to smoke, although there were some differences among sexes that require further study. Additional studies that pharmacologically prevent sleep disruption in the setting of acute and chronic exposure are needed to establish a causal role for sleep perturbation in the cardiovascular sequelae resulting from inhaled air pollution. Studies should also assess for the potential for blunted nocturnal blood pressure dipping days after exposure and impaired baroreflex function during and after exposure given previous linkages with sleep perturbation [171]. Irrespective of its exact role in triggering the cardiovascular responses in the present study, sleep perturbation, especially with repeated air pollution exposure over time, is likely to contribute to and/or worsen cardiovascular outcomes, including hypertension, particularly considering the evidence for limited habituation to poor sleep [172]. Finally, the increased frequency and magnitude of wildfires including nighttime fires and infiltration of smoke into homes during active fires may further aggravate sleep-related cardiovascular risk.

## Supplementary Information

Below is the link to the electronic supplementary material.

Supplementary Material 1.

Supplementary Material 2.

## Data Availability

All data generated or analysed during this study are included in this published article [and its supplementary information files].

## Abbreviations

ACE: Angiotensin-converting enzyme
ALP: Alkaline phosphatase
ALT: Alanine amino-transferase
ANOVA: Analysis of variance
ARV: Average real variability of blood pressure
BALF: Bronchoalveolar lavage fluid
BP: Blood pressure
BPV: Blood pressure variability
CBCs: Complete blood cell counts
CK: Creatine kinase
CO: Carbon monoxide
CO_2_: Carbon dioxide
CREB: cAMP response element-binding protein
CT: Cycle threshold
CV: Coefficient of variation of blood pressure
DBP: Diastolic blood pressure
ECG: Electrocardiogram
EDTA: Ethylenediaminetetraacetic acid
EEG: Electroencephalogram
ES: Eucalyptus smoke
FDR: False discovery rate
GGT: γ-glutamyl transferase
GLP-1: Glucagon-like peptide-1
HB: Hemoglobin
HCT: Hematocrit
HDL: High-density lipoprotein
HF: High frequency domain of heart rate variability
HR: Heart rate
HRV: Heart rate variability
IFN: Interferon
IL: Interleukin
KC-GRO: Keratinocyte chemoattractant/growth-related oncogene
LDH: Lactate dehydrogenase
LDL: Low-density lipoprotein
LF: Low frequency domain of heart rate variability
LF/HF: Ration of LF over HF
MAP: Mean arterial pressure
MCH: Mean corpuscular hemoglobin
MCHC: Mean corpuscular hemoglobin concentration
MCV: Mean corpuscular volume
MPV: Mean platelet volume
NAG: β-N-Acetylglucosaminidase
nHF: Normalized HF
nLF: Normalized LF
NREM: Non-rapid eye movement sleep
PCR: Polymerase chain reaction
PCT: Plateletcrit
PID: Proportional-integral-derivative
PLTs: Platelets
PM: Both coarse and fine and ultrafine particulate matter
PM_2.5_: Particulate matter ≤ 2.5 microns in aerodynamic diameter
PM_10_: Particulate matter ≤ 10 microns in aerodynamic diameter
PP: Pulse pressure
PYY: Peptide YY
RBCs: Red blood cells
RDW%: Red cell distribution width
REM: Rapid eye movement sleep
RMSSD: Root of the mean of squared differences of adjacent RR intervals
RT-qPCR: Reverse transcriptase real-time quantitative polymerase chain reaction
SBP: Systolic blood pressure
SD: Standard deviation of blood pressure
SDNN: Standard deviation of the RR interval
SV: Successive variation of blood pressure
TC: Total cholesterol
TG: Triglycerides
Trpa1: The gene that encodes transient receptor potential ankyrin 1
TRPA1: The cation channel expressed by trpa1
US EPA: United States Environmental Protection Agency
vLF: Very low frequency domain of heart rate variability
WBCs: White blood cells
