# Supplementary material for: Sleep disruption from inhalation of biomass smoke: a basis for coincident hypertension?
Source: Part Fibre Toxicol. 2025 Dec 11;22:34. doi: 10.1186/s12989-025-00650-9 (PMC12696922; doi:10.1186/s12989-025-00650-9)
Supplement: Supplementary file 2 — Supplementary Material 2. [file 12989_2025_650_MOESM2_ESM.docx]

**Rentschler et al Supplementary Information**

**Supplementary Tables**

**Table S1**: Correlation analysis of LF/HF vs. cardiovascular endpoints during exposure.

| LF/HF vs. Cardiovascular Endpoints | | | | |
| --- | --- | --- | --- | --- |
|  | Female-Air | Female-Smoke | Male-Air | Male-Smoke |
| Heart Rate (bpm) | r^2^=0.1986; p=0.1466 | r^2^=0.4267; p=0.0213* | r^2^=0.1426; p=0.2262 | r^2^=0.5312; p=0.0072* |
| Systolic BP (mmHg) | r^2^=0.1793; p=0.1701 | r^2^=0.2820; p=0.0757 | r^2^=0.3731; p=0.0349 | r^2^=0.5944; p=0.0033* |
| Diastolic BP (mmHg) | r^2^=0.1794; p=0.1701 | r^2^=0.2331; p=0.1119 | r^2^=0.3455; p=0.0444* | r^2^=0.5095; p=0.0091* |
| Pulse Pressure (mmHg) | r^2^=0.1324; p=0.2450 | r^2^=0.2478; p=0.0996 | r^2^=0.1248; p=0.2600 | r^2^=0.3239; p=0.0534 |
| MAP (mmHg) | r^2^=0.1811; p=0.1679 | r^2^=0.2479; p=0.0995 | r^2^=0.3055; p=0.0623 | r^2^=0.5546; p=0.0055* |

Correlations were derived from plots of five-minute averages of LF/HF vs. concurrent measures of cardiovascular physiology per 5-minute period for each animal over the course of 1-hr of exposure (12 values per animal).

**Table S2**: Correlation analysis of sleep parameters vs. a frequency domain measure of HRV during exposure.

|  | Female-Air | Female-Smoke | Male-Air | Male-Smoke |
| --- | --- | --- | --- | --- |
| NREM vs. LF/HF | r^2^=0.3403; p=0.0465 | r^2^=0.3432; p=0.0453* | r^2^=0.1536; p=0.2077 | r^2^=0.2861; p=0.0732 |
| Wake vs. LF/HF | r^2^=0.2764; p=0.0792 | r^2^=0.3971; p=0.0281* | r^2^=0.1176; p=0.2752 | r^2^=0.2989; p=0.0659 |

Correlations were derived from plots of five-minute averages of NREM or wake duration vs. concurrent measures of LF/HF per 5-minute period for each animal over the course of 1-hr of exposure (12 values per animal).

**Table S3**: Correlation analysis of sleep parameters vs. cardiovascular endpoints after exposure.

| NREM Duration (min) vs. Cardiovascular Endpoints | | | | |
| --- | --- | --- | --- | --- |
|  | Female-Air | Female-Smoke | Male-Air | Male-Smoke |
| Heart Rate (bpm) | r^2^=0.8141; p=0.0022* | r^2^=0.6219; p=0.0200* | r^2^=0.8042; p=0.0025* | r^2^=0.8260; p=0.0018* |
| Systolic BP (mmHg) | r^2^=0.6201; p=0.0203* | r^2^=0.3084; p=0.1530 | r^2^=0.5124; p=0.0458* | r^2^=0.8063; p=0.0025* |
| Diastolic BP (mmHg) | r^2^=0.6969; p=0.0099* | r^2^=0.4635; p=0.0631 | r^2^=0.5684; p=0.0307* | r^2^=0.8328; p=0.0016* |
| Pulse Pressure (mmHg) | r^2=^0.2314; p=0.2275 | r^2^=0.0306; p=0.6788 | r^2^=0.2152; p=0.2570 | r^2^=0.2619; p=0.1948 |
| MAP (mmHg) | r^2^=0.6859; p=0.0111* | r^2^=0.3848; p=0.1008 | r^2^=0.5572; p=0.0334* | r^2^=0.8378; p=0.0014* |
| Wake Duration (min) vs. Cardiovascular Endpoints | | | | |
|  | Female-Air | Female-Smoke | Male-Air | Male-Smoke |
| Heart Rate (bpm) | r^2^=0.8998; p=0.0003* | r^2^=0.5893; p=0.0261* | r^2^=0.7109; p=0.0085* | r^2^=0.7723; p=0.0041* |
| Systolic BP (mmHg) | r^2^=0.8505; p=0.0011* | r^2^=0.2270; p=0.2327 | r^2^=0.5353; p=0.0391* | r^2^=0.7552; p=0.0051* |
| Diastolic BP (mmHg) | r^2^=0.9070; p=0.0003* | r^2^=0.3594; p=0.1162 | r^2^=0.6154; p=0.0212* | r^2^=0.8039; p=0.0026* |
| Pulse Pressure (mmHg) | r^2^=0.3460; p=0.1251 | r^2^=0.0103; p=0.8110 | r^2^=0.1747; p=0.3027 | r^2^=0.1854; p=0.2869 |
| MAP (mmHg) | r^2^=0.8982; p=0.0003* | r^2^=0.2987; p=0.1618 | r^2^=0.6075; p=0.0226* | r^2^=0.7985; p=0.0028* |

Correlations were derived from plots of 1-hr averages of NREM or wake duration vs. concurrent measures of cardiovascular physiology over the course of the post-exposure period (8 values per animal).

**Table S4**: Correlation analysis of frequency domain measures of HRV vs. cardiovascular endpoints after exposure.

| nLF vs. Cardiovascular Endpoints | | | | |
| --- | --- | --- | --- | --- |
|  | Female-Air | Female-Smoke | Male-Air | Male-Smoke |
| Heart Rate (bpm) | r^2^=0.2949; p=0.1643 | r^2^=0.8203; p=0.0019* | r^2^=0.6527; p=0.0153* | r^2^=0.6791; p=0.0019* |
| Systolic BP (mmHg) | r^2^=0.2482; p=0.2090 | r^2^=0.4395; p=0.0731 | r^2^=0.2272; p=0.2325 | r^2^=0.5143; p=0.0452* |
| Diastolic BP (mmHg) | r^2^=0.3953; p=0.0950 | r^2^=0.5608; p=0.0325* | r^2^=0.4082; p=0.0881 | r^2^=0.5286; p=0.0410* |
| Pulse Pressure (mmHg) | r^2^=0.0439; p=0.6185 | r^2^=0.1083; p=0.4261 | r^2^=0.0004; p=0.9627 | r^2^=0.1607; p=0.3251 |
| MAP (mmHg) | r^2^=0.2822; p=0.1755 | r^2^=0.5058; p=0.0479* | r^2^=0.3506; p=0.1220 | r^2^=0.5385; p=0.0382* |
| LF/HF vs. Cardiovascular Endpoints | | | | |
|  | Female-Air | Female-Smoke | Male-Air | Male-Smoke |
| Heart Rate (bpm) | r^2^=0.3193; p=0.1445 | r^2^=0.5383; p=0.0383* | r^2^=0.5617; p=0.0323* | r^2^=0.7074; p=0.0089* |
| Systolic BP (mmHg) | r^2^=0.3089; p=0.1526 | r^2^=0.2190; p=0.2423 | r^2^=0.1395; p=0.3620 | r^2^=0.4787; p=0.0573 |
| Diastolic BP (mmHg) | r^2^=0.4777; p=0.0576 | r^2^=0.3512; p=0.1216 | r^2^=0.2941; p=0.1650 | r^2^=0.4749; p=0.0587 |
| Pulse Pressure (mmHg) | r^2^=0.0555; p=0.5742 | r^2^=0.0098; p=0.8157 | r^2^=0.0110; p=0.8052 | r^2^=0.2033; p=0.2621 |
| MAP (mmHg) | r^2^=0.3506; p=0.1220 | r^2^=0.2919; p=0.1668 | r^2^=0.2442; p=0.2132 | r^2^=0.4948; p=0.0516 |

Correlations were derived from plots of 1-hour averages of nLF or LF/HF vs. concurrent measures of cardiovascular physiology over the course of the post-exposure period (8 values per animal).

**Table S5**: Correlation analysis of sleep parameters vs. frequency domain measures of HRV after exposure.

| Sleep parameters vs. nLF | | | | |
| --- | --- | --- | --- | --- |
|  | Female-Air | Female-Smoke | Male-Air | Male-Smoke |
| NREM duration (min) | r^2^=0.2157; p=0.2463 | r^2^=0.8897; p=0.0004* | r^2^=0.4843; p=0.0552 | r^2^=0.7323; p=0.0067* |
| Wake duration (min) | r^2^=0.4059; p=0.0893 | r^2^=0.8406; p=0.0013* | r^2^=0.7080; p=0.0088* | r^2^=0.8251; p=0.0018* |
| Sleep parameters vs. LF/HF | | | | |
|  | Female-Air | Female-Smoke | Male-Air | Male-Smoke |
| NREM duration (min) | r^2^=0.2087; p=0.2552 | r^2^=0.8983; p=0.0003* | r^2^=0.4012; p=0.0918 | r^2^=0.6793; p=0.0119* |
| Wake duration (min) | r^2^=0.4441; p=0.0711 | r^2^=0.9802; p<0.0001* | r^2^=0.6233; p=0.0198* | r^2^=0.7851; p=0.0034* |

Correlations were derived from plots of 1-hour averages of nLF or LF/HF vs. concurrent measures of sleep over the course of the post-exposure period (8 values per animal).

**Table S6**: Bronchoalveolar lavage fluid (BALF), whole blood, and serum factors one day after exposure in female and male rats.

| Group | Female Filtered Air | Female Smoke | Male Filtered Air | Male Smoke |
| --- | --- | --- | --- | --- |
| BALF Total Cells (cells/ml) | 47010±10774 | 46153±9395 | 39480±14311 | 41351±17153 |
| BALF Macrophages (cells/ml) | 42419±9541 | 40666±8514 | 34362±12129 | 37226±16072 |
| BALF Neutrophils (cells/ml) | 1795±967 | 3028±997^a^ | 2610±2142 | 1912±801 |
| BALF Eosinophils (cells/ml) | 136±231 | 141±189 | 603±1296 | 28±78 |
| BALF Lymphocytes (cells/ml) | 708±440 | 628±209 | 694±353 | 823±371 |
| BALF Albumin (mg/L) | 29.95±8.81 | 26.80±9.69 | 20.28±7.41 | 15.16±2.66 |
| BALF Protein (μg/ml) | 96.06±15.22 | 94.72±24.95 | 73.74±26.87 | 60.50±10.34 |
| BALF GGT (U/I) | 6.92±0.25 | 6.39±0.12^b^ | 6.05±0.65 | 6.08±1.18 |
| BALF LDH (U/I) | 23.83±3.13 | 21.02±5.01 | 13.17±2.91 | 10.97±0.91 |
| BALF NAG (U/I) | 9.23±0.85 | 8.35±0.23^b^ | 7.72±0.26 | 7.57±0.18 |
| Whole Blood WBC (K/μl) | 4.66±1.22 | 6.67±2.20^a^ | 7.29±2.44 | 7.37±1.12 |
| Whole Blood Neutrophils (K/μl) | 0.82±0.23 | 1.11±0.35 | 1.65±0.64 | 1.92±0.38 |
| Whole Blood Lymphocytes (K/μl) | 3.68±1.09 | 5.38±1.91^c^ | 5.40±1.76 | 5.21±1.04 |
| Whole Blood Monocytes (K/μl) | 0.15±0.06 | 0.18±0.07 | 0.23±0.12 | 0.21±0.08 |
| Whole Blood Eosinophils (K/μl) | 0.0063±0.0052 | 0.0083±0.0075 | 0.013±0.010 | 0.019±0.018 |
| Whole Blood RBC (M/μl) | 7.73±0.32 | 7.52±0.36 | 8.49±0.42 | 7.95±0.29^b^ |
| Whole Blood HB (g/dL) | 15.70±0.50 | 15.50±0.59 | 16.93±0.69 | 16.79±0.68 |
| Whole Blood HCT (%) | 49.69±2.51 | 48.38±2.96 | 53.09±2.71 | 51.30±2.64 |
| Whole Blood MCV (fL) | 64.28±2.15 | 64.32±2.08 | 62.65±4.04 | 64.56±2.15 |
| Whole Blood MCH (pg) | 20.33±0.51 | 20.60±1.11 | 19.96±1.11 | 21.13±0.82^a^ |
| Whole Blood MCHC (g/dL) | 31.61±0.69 | 32.08±0.91 | 31.90±0.78 | 32.75±0.79^a^ |
| Whole Blood RDW (%) | 13.99±0.44 | 14.12±0.82 | 15.35±1.43 | 14.95±0.60 |
| Whole Blood PLT (K/μl) | 966±111 | 908±97 | 950±102 | 959±85 |
| Whole Blood PCT (%) | 0.558±76 | 0.533±60 | 0.585±72 | 0.581±52 |
| Whole Blood MPV (fL) | 5.76±0.18 | 5.88±0.25 | 6.15±0.25 | 6.06±0.28 |
| Serum CRP (mg/dl) | 0.094±0.049 | 0.108±0.064 | 0.081±0.060 | 0.173±0.042^a^ |
| Serum Total Chol. (mg/dl) | 45.29±5.70 | 43.72±2.98 | 42.48±2.71 | 45.08±6.99 |
| Serum LDL Chol. (mg/dl)) | 19.41±2.79 | 18.45±1.42 | 17.13±1.93 | 16.96±1.98 |
| Serum HDL Chol. (mg/dl) | 6.23±1.00 | 6.18±1.23 | 8.16±1.80 | 8.47±1.97 |
| Serum Triglycerides (mg/dl) | 72.95±39.32 | 53.85±31.34 | 111.3±63.84 | 123.4±76.80 |
| Serum FFA (μM) | 359±169 | 243±86 | 357±162 | 343±117 |
| Serum Glucose (mg/dl) | 253±33 | 238±24 | 247±26 | 252±19 |
| Serum ACE (U/I) | 159±34 | 168±28 | 226±68 | 223±54 |
| Serum ALP (U/I) | 24.53±3.15 | 31.92±3.83^a^ | 28.77±2.29 | 27.53±5.85 |
| Serum ALT (U/I) | 51.24±9.30 | 64.33±17.04 | 55.45±12.80 | 62.39±15.30 |
| Serum C3 (mg/dl) | 32.20±4.26 | 31.08±1.70 | 32.07±3.55 | 34.20±6.42 |
| Serum C4 (mg/dl) | 4.32±0.74 | 3.94±0.48 | 3.13±1.10 | 3.58±0.73 |
| Serum CK (U/I) | 557±430 | 413±382 | 487±305 | 287±137 |
| Serum IFN-γ (pg/ml) | 5.35±1.95 | 6.62±1.50 | 4.94±1.95 | 7.43±2.33^a^ |
| Serum IL-4 (pg/ml) | 1.60±0.20 | 1.65±0.29 | 1.40±0.32 | 1.94±0.25^a^ |
| Serum IL-6 (pg/ml) | 11.46±3.23 | 14.00±3.45 | 18.77±4.42 | 19.51±4.05 |
| Serum IL-10 (pg/ml) | 24.96±3.61 | 31.30±1.91^a^ | 26.44±4.71 | 34.82±4.29^a^ |
| Serum IL-13 (pg/ml) | 2.73±0.60 | 3.07±0.53 | 4.01±1.22 | 4.18±0.89 |
| Serum KC-GRO (pg/ml) | 242±95 | 241±39 | 181±73 | 178±41 |
| Serum TNF-α (pg/ml) | 2.31±0.53 | 3.05±0.50^a^ | 3.11±0.65 | 2.76±0.58 |
| Serum C-peptide (pg/ml) | 8015±1697 | 7827±3605 | 7580±3648 | 9329±3251 |
| Serum GLP-1 (pmol/ml) | 9.68±2.34 | 10.0±2.52 | 12.64±5.33 | 13.90±4.79 |
| Serum PYY (pg/ml) | 195±30 | 182±42 | 185±22 | 202±35 |
| Serum Glucagon (pmol/ml) | 13.49±8.09 | 12.76±2.27 | 15.19±7.53 | 19.22±8.52 |
| Serum Insulin (μIU/ml) | 147.00±54.91 | 213±116 | 152.60±93.37 | 217.60±77.79 |
| Serum Leptin (pg/ml) | 9522±3590 | 17286±17329 | 15395±5957 | 17780±5551 |

Values represent means ± standard deviation (n=8/group for all groups except female-smoke, which had an n=6, except for serum IFN- γ, IL-4, IL-6, IL-10, IL-13, KC-GRO, TNF- α, C-peptide, GLP-1, PYY, glucagon, insulin and leptin, which had an n=5, and IL-10 and insulin, which had an n=4 (one value was below the limit of detection)). BALF = bronchoalveolar lavage fluid; GGT- gamma glutamyl transferase; LDH = lactate dehydrogenase; NAG = N-acetyl glucosaminidase; WBC = white blood cell count, RBC = red blood cell count, HB = hemoglobin; HCT = hematocrit; MCV = mean corpuscular volume; MCH = mean corpuscular hemoglobin; MCHC = mean corpuscular hemoglobin concentration; RDW (%) – red blood distribution width; PLT = platelet count; PCT – plateletcrit; MPV = mean platelet volume. Chol. = cholesterol; LDL = low density lipoprotein; HDL = high density lipoprotein; ACE = angiotensin converting enzyme; ALP = alkaline phosphatase; ALT = alanine aminotransferase; C3 and C4 = complement components 3 and 4; CK = creatine kinase; IFN = interferon; IL = interleukin; KC-GRO = keratinocyte chemoattractant/growth regulated oncogene; TNF = tumor necrosis factor; GLP-1 = glucagon-like peptide-1; PYY = peptide YY. a – significantly greater than filtered air control of the same sex; b – significantly less than filtered air control of the same sex, c= p=0.0563 vs. filtered air control of the same sex. Note: Serum IL-1β and IL-5 levels were not listed in the above table because they were below limits of detection. Also, there were no basophils detected in whole blood.

**Table S7**: Fold-differences in gene expression in the hypothalamic tissue of females.

| Filtered Air | | | | | | Eucalyptus Smoke | | | | | |
| --- | --- | --- | --- | --- | --- | --- | --- | --- | --- | --- | --- |
|  |  |  |  |  |  |  |  |  |  |  |  |
| Gene | Mean | Std Error | N | Coeff of Variation |  | Mean | Std Error | N | Coeff of Variation | t-test | FDR q-value |
| Aanat | 1.00 | 0.12 | 8 | 33.7 |  | 0.97 | 0.10 | 4 | 20.9 | 0.97579 | 1.000 |
| Alas1 | 1.00 | 0.03 | 8 | 7.2 |  | 0.96 | 0.05 | 4 | 10.3 | 0.40943 | 0.962 |
| Arntl | 1.00 | 0.02 | 8 | 6.6 |  | 0.96 | 0.08 | 4 | 17.3 | 0.49968 | 0.962 |
| Arntl2 | 1.00 | 0.10 | 8 | 28.8 |  | 1.03 | 0.09 | 4 | 16.9 | 0.73589 | 1.000 |
| Atoh7 | 1.00 | 0.08 | 8 | 21.2 |  | 1.13 | 0.15 | 4 | 25.8 | Unexpressed | |
| Bhlhe40 | 1.00 | 0.01 | 8 | 4.1 |  | 1.05 | 0.04 | 4 | 6.9 | 0.14924 | 0.810 |
| Bhlhe41 | 1.00 | 0.04 | 8 | 11.4 |  | 1.00 | 0.02 | 4 | 4.3 | 0.9618 | 1.000 |
| Camk2a | 1.00 | 0.06 | 8 | 17.1 |  | 1.11 | 0.09 | 4 | 15.4 | 0.30617 | 0.962 |
| Camk2b | 1.00 | 0.03 | 8 | 9.7 |  | 1.03 | 0.01 | 4 | 1.3 | 0.59469 | 0.996 |
| Camk2d | 1.00 | 0.03 | 8 | 9.8 |  | 1.08 | 0.02 | 4 | 3.0 | 0.16027 | 0.810 |
| Camk2g | 1.00 | 0.04 | 8 | 11.4 |  | 1.07 | 0.03 | 4 | 4.8 | 0.32173 | 0.962 |
| Cartpt | 1.00 | 0.07 | 8 | 18.8 |  | 0.91 | 0.10 | 4 | 22.4 | 0.4566 | 0.962 |
| Ccrn4l | 1.00 | 0.03 | 8 | 8.5 |  | 0.99 | 0.06 | 4 | 11.5 | 0.86944 | 1.000 |
| Chrnb2 | 1.00 | 0.04 | 8 | 11.4 |  | 0.97 | 0.10 | 4 | 19.8 | 0.65678 | 0.996 |
| Clock | 1.00 | 0.04 | 8 | 11.3 |  | 1.01 | 0.04 | 4 | 7.4 | 0.81837 | 1.000 |
| Creb1 | 1.00 | 0.04 | 8 | 10.7 |  | 1.05 | 0.02 | 4 | 4.2 | 0.37213 | 0.962 |
| Creb3 | 1.00 | 0.03 | 8 | 7.4 |  | 0.99 | 0.02 | 4 | 4.9 | 0.91306 | 1.000 |
| Crx | 1.00 | 0.09 | 8 | 25.5 |  | 0.89 | 0.07 | 4 | 15.8 | Unexpressed | |
| Cry1 | 1.00 | 0.04 | 8 | 11.5 |  | 0.83 | 0.05 | 4 | 11.5 | 0.0294 | 0.792 |
| Cry2 | 1.00 | 0.03 | 8 | 8.1 |  | 1.05 | 0.04 | 4 | 7.8 | 0.38608 | 0.962 |
| Csnk1a1 | 1.00 | 0.04 | 8 | 11.5 |  | 1.01 | 0.02 | 4 | 4.5 | 0.75279 | 1.000 |
| Csnk1d | 1.00 | 0.02 | 8 | 7.0 |  | 0.98 | 0.05 | 4 | 10.3 | 0.6203 | 0.996 |
| Csnk1e | 1.00 | 0.03 | 8 | 9.7 |  | 1.00 | 0.05 | 4 | 9.3 | 0.92709 | 1.000 |
| Csnk2a1 | 1.00 | 0.03 | 8 | 8.1 |  | 1.08 | 0.03 | 4 | 6.0 | 0.1128 | 0.810 |
| Csnk2a2 | 1.00 | 0.02 | 8 | 6.5 |  | 1.06 | 0.03 | 4 | 5.6 | 0.13644 | 0.810 |
| Dbp | 1.00 | 0.04 | 8 | 12.2 |  | 0.94 | 0.07 | 4 | 15.6 | 0.47848 | 0.962 |
| Egr1 | 1.00 | 0.06 | 8 | 18.4 |  | 0.91 | 0.13 | 4 | 28.9 | 0.44651 | 0.962 |
| Egr3 | 1.00 | 0.12 | 8 | 34.7 |  | 1.27 | 0.14 | 4 | 22.5 | 0.1585 | 0.810 |
| Epo | 1.00 | 0.03 | 8 | 8.9 |  | 1.08 | 0.10 | 4 | 19.1 | Unexpressed | |
| Esrra | 1.00 | 0.06 | 8 | 17.4 |  | 1.02 | 0.13 | 4 | 25.0 | 0.9388 | 1.000 |
| Fbxl3 | 1.00 | 0.03 | 8 | 7.8 |  | 0.96 | 0.04 | 4 | 8.7 | 0.43702 | 0.962 |
| Hebp1 | 1.00 | 0.02 | 8 | 6.1 |  | 0.95 | 0.04 | 4 | 8.7 | 0.2554 | 0.962 |
| Hlf | 1.00 | 0.05 | 8 | 14.6 |  | 0.94 | 0.07 | 4 | 15.7 | 0.49165 | 0.962 |
| Htr7 | 1.00 | 0.03 | 8 | 8.3 |  | 1.08 | 0.03 | 4 | 6.4 | 0.12689 | 0.810 |
| Irf1 | 1.00 | 0.05 | 8 | 13.0 |  | 1.03 | 0.07 | 4 | 13.4 | 0.68523 | 0.996 |
| Kcnma1 | 1.00 | 0.02 | 8 | 6.9 |  | 1.01 | 0.07 | 4 | 14.2 | 0.9121 | 1.000 |
| Mapk1 | 1.00 | 0.01 | 8 | 4.1 |  | 0.97 | 0.03 | 4 | 5.3 | 0.36213 | 0.962 |
| Mapk14 | 1.00 | 0.03 | 8 | 7.2 |  | 1.09 | 0.05 | 4 | 9.8 | 0.12338 | 0.810 |
| Mapk3 | 1.00 | 0.02 | 8 | 5.0 |  | 1.01 | 0.05 | 4 | 9.2 | 0.76091 | 1.000 |
| Mat2a | 1.00 | 0.04 | 8 | 10.0 |  | 1.06 | 0.07 | 4 | 12.5 | 0.44837 | 0.962 |
| Mtnr1a | 1.00 | 0.13 | 8 | 35.4 |  | 0.59 | 0.13 | 4 | 43.4 | 0.0697 | 0.810 |
| Mtnr1b | 1.00 | 0.15 | 8 | 41.9 |  | 0.77 | 0.11 | 4 | 28.7 | 0.43478 | 0.962 |
| Myod1 | 1.00 | 0.15 | 8 | 43.0 |  | 0.88 | 0.22 | 4 | 49.9 | 0.64252 | 0.996 |
| Ncoa3 | 1.00 | 0.04 | 8 | 11.4 |  | 0.96 | 0.03 | 4 | 6.6 | 0.55631 | 0.996 |
| Nfil3 | 1.00 | 0.06 | 8 | 16.8 |  | 0.89 | 0.12 | 4 | 26.0 | 0.29108 | 0.962 |
| Nkx2-5 | 1.00 | 0.03 | 8 | 7.6 |  | 1.05 | 0.10 | 4 | 19.1 | Unexpressed | |
| Nms | 1.00 | 0.21 | 8 | 59.9 |  | 1.16 | 0.12 | 4 | 20.6 | 0.42392 | 0.962 |
| Npas2 | 1.00 | 0.03 | 8 | 8.9 |  | 1.08 | 0.10 | 4 | 19.1 | Unexpressed | |
| Nr1d1 | 1.00 | 0.06 | 8 | 15.6 |  | 0.91 | 0.11 | 4 | 25.2 | 0.38035 | 0.962 |
| Nr1d2 | 1.00 | 0.02 | 8 | 5.6 |  | 0.93 | 0.04 | 4 | 9.0 | 0.11603 | 0.810 |
| Nr2f6 | 1.00 | 0.04 | 8 | 9.9 |  | 1.03 | 0.05 | 4 | 10.5 | 0.66579 | 0.996 |
| Opn3 | 1.00 | 0.04 | 8 | 12.7 |  | 1.11 | 0.04 | 4 | 6.7 | 0.15935 | 0.810 |
| Opn4 | 1.00 | 0.07 | 8 | 21.0 |  | 1.48 | 0.14 | 4 | 18.6 | 0.00902 | 0.365 |
| Pax4 | 1.00 | 0.07 | 8 | 20.3 |  | 1.19 | 0.14 | 4 | 23.4 | 0.20473 | 0.920 |
| Per1 | 1.00 | 0.09 | 8 | 25.5 |  | 0.96 | 0.07 | 4 | 15.3 | 0.86831 | 1.000 |
| Per2 | 1.00 | 0.06 | 8 | 17.9 |  | 0.98 | 0.05 | 4 | 10.6 | 0.91941 | 1.000 |
| Per3 | 1.00 | 0.03 | 8 | 9.5 |  | 0.89 | 0.07 | 4 | 15.0 | 0.09846 | 0.810 |
| Pou2f1 | 1.00 | 0.03 | 8 | 9.0 |  | 1.01 | 0.06 | 4 | 11.0 | 0.91231 | 1.000 |
| Ppara | 1.00 | 0.05 | 8 | 15.0 |  | 0.97 | 0.04 | 4 | 9.1 | 0.8068 | 1.000 |
| Ppargc1a | 1.00 | 0.05 | 8 | 13.4 |  | 1.00 | 0.07 | 4 | 14.5 | 0.97867 | 1.000 |
| Prf1 | 1.00 | 0.10 | 8 | 27.0 |  | 1.22 | 0.37 | 4 | 60.5 | Unexpressed | |
| Prkaca | 1.00 | 0.03 | 8 | 8.1 |  | 0.97 | 0.05 | 4 | 9.9 | 0.53379 | 0.996 |
| Prkacb | 1.00 | 0.04 | 8 | 12.7 |  | 0.95 | 0.05 | 4 | 11.6 | 0.48866 | 0.962 |
| Prkar1a | 1.00 | 0.03 | 8 | 9.7 |  | 1.02 | 0.01 | 4 | 2.7 | 0.69009 | 0.996 |
| Prkar1b | 1.00 | 0.04 | 8 | 10.0 |  | 1.02 | 0.02 | 4 | 4.7 | 0.68858 | 0.996 |
| Prkar2a | 1.00 | 0.03 | 8 | 7.9 |  | 0.90 | 0.07 | 4 | 15.7 | 0.15594 | 0.810 |
| Prkar2b | 1.00 | 0.02 | 8 | 5.5 |  | 1.00 | 0.02 | 4 | 3.9 | 0.92075 | 1.000 |
| Prkca | 1.00 | 0.03 | 8 | 9.4 |  | 1.03 | 0.04 | 4 | 8.0 | 0.61577 | 0.996 |
| Prkcb | 1.00 | 0.02 | 8 | 5.0 |  | 1.03 | 0.01 | 4 | 1.8 | 0.35927 | 0.962 |
| Prokr2 | 1.00 | 0.05 | 8 | 12.9 |  | 1.07 | 0.16 | 4 | 29.3 | 0.68516 | 0.996 |
| Ptgds | 1.00 | 0.19 | 8 | 53.8 |  | 0.99 | 0.26 | 4 | 52.0 | 0.90441 | 1.000 |
| Rora | 1.00 | 0.03 | 8 | 8.2 |  | 1.00 | 0.01 | 4 | 2.7 | 0.97942 | 1.000 |
| Rorb | 1.00 | 0.04 | 8 | 11.0 |  | 0.94 | 0.05 | 4 | 10.7 | 0.39973 | 0.962 |
| Rorc | 1.00 | 0.11 | 8 | 30.8 |  | 0.72 | 0.07 | 4 | 19.3 | 0.11644 | 0.810 |
| Slc9a3 | 1.00 | 0.10 | 8 | 27.8 |  | 1.65 | 0.19 | 4 | 23.1 | 0.00771 | 0.365 |
| Smad4 | 1.00 | 0.02 | 8 | 4.8 |  | 0.97 | 0.05 | 4 | 10.8 | 0.4113 | 0.962 |
| Sp1 | 1.00 | 0.03 | 8 | 9.3 |  | 0.95 | 0.05 | 4 | 10.6 | 0.36056 | 0.962 |
| Srebf1 | 1.00 | 0.05 | 8 | 15.5 |  | 0.94 | 0.11 | 4 | 23.8 | 0.54651 | 0.996 |
| Stat5a | 1.00 | 0.09 | 8 | 25.1 |  | 0.98 | 0.09 | 4 | 18.7 | 0.99841 | 1.000 |
| Tcfap2a | 1.00 | 0.09 | 8 | 25.4 |  | 1.00 | 0.09 | 4 | 19.0 | Unexpressed | |
| Tef | 1.00 | 0.03 | 8 | 9.7 |  | 0.99 | 0.08 | 4 | 15.5 | 0.83614 | 1.000 |
| Tgfb1 | 1.00 | 0.09 | 8 | 24.1 |  | 0.92 | 0.09 | 4 | 20.2 | 0.60746 | 0.996 |
| Timeless | 1.00 | 0.17 | 8 | 48.0 |  | 0.67 | 0.12 | 4 | 35.5 | 0.32626 | 0.962 |
| Wee1 | 1.00 | 0.02 | 8 | 6.9 |  | 0.95 | 0.02 | 4 | 4.8 | 0.18831 | 0.896 |

**Table S8**: Fold-differences in gene expression in the hypothalamic tissue of males.

| Filtered Air | | | | | | Eucalyptus Smoke | | | | | |
| --- | --- | --- | --- | --- | --- | --- | --- | --- | --- | --- | --- |
|  |  |  |  |  |  |  |  |  |  |  |  |
| Variable | Mean | Std Error | N | Coeff of Variation |  | Mean | Std Error | N | Coeff of Variation | t-test | FDR q-value |
| Aanat | 1.00 | 0.12 | 8 | 33.3 |  | 1.01 | 0.07 | 8 | 20.3 | 0.7446 | 0.9259 |
| Alas1 | 1.00 | 0.06 | 8 | 18.2 |  | 0.89 | 0.04 | 8 | 11.8 | 0.14786 | 0.7260 |
| Arntl | 1.00 | 0.04 | 8 | 11.3 |  | 0.94 | 0.02 | 8 | 6.3 | 0.25378 | 0.7260 |
| Arntl2 | 1.00 | 0.09 | 8 | 24.6 |  | 1.08 | 0.16 | 8 | 41.5 | 0.89533 | 0.9907 |
| Atoh7 | 1.00 | 0.13 | 8 | 37.6 |  | 0.71 | 0.03 | 8 | 12.8 | Unexpressed | |
| Bhlhe40 | 1.00 | 0.06 | 8 | 17.0 |  | 0.95 | 0.05 | 8 | 14.4 | 0.50778 | 0.8046 |
| Bhlhe41 | 1.00 | 0.02 | 8 | 6.8 |  | 0.92 | 0.02 | 8 | 5.1 | 0.01869 | 0.4785 |
| Camk2a | 1.00 | 0.03 | 8 | 8.8 |  | 1.00 | 0.05 | 8 | 14.1 | 0.93376 | 1.0000 |
| Camk2b | 1.00 | 0.04 | 8 | 12.3 |  | 0.94 | 0.03 | 8 | 8.2 | 0.32488 | 0.7260 |
| Camk2d | 1.00 | 0.04 | 8 | 12.5 |  | 0.92 | 0.03 | 8 | 8.5 | 0.13456 | 0.7260 |
| Camk2g | 1.00 | 0.04 | 8 | 12.4 |  | 0.98 | 0.02 | 8 | 5.0 | 0.8148 | 0.9664 |
| Cartpt | 1.00 | 0.09 | 8 | 26.6 |  | 0.93 | 0.04 | 8 | 11.5 | 0.6678 | 0.8954 |
| Ccrn4l | 1.00 | 0.04 | 8 | 11.0 |  | 0.92 | 0.04 | 8 | 13.0 | 0.13645 | 0.7260 |
| Chrnb2 | 1.00 | 0.08 | 8 | 23.9 |  | 0.89 | 0.07 | 8 | 22.4 | 0.40415 | 0.7770 |
| Clock | 1.00 | 0.04 | 8 | 11.9 |  | 0.97 | 0.02 | 8 | 5.6 | 0.60368 | 0.8895 |
| Creb1 | 1.00 | 0.04 | 8 | 12.1 |  | 0.97 | 0.03 | 8 | 7.9 | 0.62261 | 0.8895 |
| Creb3 | 1.00 | 0.02 | 8 | 6.5 |  | 0.96 | 0.03 | 8 | 8.0 | 0.2662 | 0.7260 |
| Crx | 1.00 | 0.15 | 8 | 41.6 |  | 0.86 | 0.08 | 8 | 26.0 | Unexpressed | |
| Cry1 | 1.00 | 0.04 | 8 | 11.8 |  | 0.88 | 0.07 | 8 | 21.3 | 0.13154 | 0.7260 |
| Cry2 | 1.00 | 0.06 | 8 | 17.5 |  | 0.96 | 0.02 | 8 | 5.7 | 0.68578 | 0.9055 |
| Csnk1a1 | 1.00 | 0.04 | 8 | 10.8 |  | 0.99 | 0.03 | 8 | 9.4 | 0.89803 | 0.9907 |
| Csnk1d | 1.00 | 0.06 | 8 | 16.8 |  | 0.91 | 0.04 | 8 | 12.7 | 0.31868 | 0.7260 |
| Csnk1e | 1.00 | 0.04 | 8 | 12.3 |  | 0.95 | 0.04 | 8 | 10.8 | 0.41628 | 0.7770 |
| Csnk2a1 | 1.00 | 0.05 | 8 | 14.5 |  | 0.96 | 0.01 | 8 | 3.6 | 0.57007 | 0.8872 |
| Csnk2a2 | 1.00 | 0.03 | 8 | 7.4 |  | 0.95 | 0.02 | 8 | 6.9 | 0.2371 | 0.7260 |
| Dbp | 1.00 | 0.06 | 8 | 17.8 |  | 0.93 | 0.02 | 8 | 7.0 | 0.36936 | 0.7316 |
| Egr1 | 1.00 | 0.07 | 8 | 20.7 |  | 0.79 | 0.10 | 8 | 35.3 | 0.08831 | 0.6997 |
| Egr3 | 1.00 | 0.04 | 8 | 11.0 |  | 0.74 | 0.02 | 8 | 9.3 | 0.00004 | 0.0035* |
| Epo | 1.00 | 0.07 | 8 | 20.6 |  | 0.90 | 0.04 | 8 | 11.9 | Unexpressed | |
| Esrra | 1.00 | 0.16 | 8 | 45.6 |  | 0.82 | 0.09 | 8 | 32.1 | 0.35207 | 0.7306 |
| Fbxl3 | 1.00 | 0.06 | 8 | 16.2 |  | 0.92 | 0.03 | 8 | 9.7 | 0.31133 | 0.7260 |
| Hebp1 | 1.00 | 0.05 | 8 | 12.9 |  | 0.87 | 0.04 | 8 | 12.2 | 0.05278 | 0.6250 |
| Hlf | 1.00 | 0.12 | 8 | 32.5 |  | 0.90 | 0.06 | 8 | 18.6 | 0.50384 | 0.8046 |
| Htr7 | 1.00 | 0.05 | 8 | 14.7 |  | 0.94 | 0.05 | 8 | 13.9 | 0.42171 | 0.7770 |
| Irf1 | 1.00 | 0.05 | 8 | 15.1 |  | 0.99 | 0.07 | 8 | 19.4 | 0.84565 | 0.9664 |
| Kcnma1 | 1.00 | 0.06 | 8 | 15.6 |  | 0.92 | 0.03 | 8 | 8.5 | 0.30085 | 0.7260 |
| Mapk1 | 1.00 | 0.02 | 8 | 6.5 |  | 0.94 | 0.02 | 8 | 5.6 | 0.05369 | 0.6250 |
| Mapk14 | 1.00 | 0.03 | 8 | 9.6 |  | 0.99 | 0.03 | 8 | 9.5 | 0.84748 | 0.9664 |
| Mapk3 | 1.00 | 0.06 | 8 | 15.9 |  | 0.92 | 0.03 | 8 | 8.5 | 0.24585 | 0.7260 |
| Mat2a | 1.00 | 0.05 | 8 | 14.9 |  | 1.05 | 0.04 | 8 | 11.2 | 0.45876 | 0.7770 |
| Mtnr1a | 1.00 | 0.12 | 8 | 32.8 |  | 1.05 | 0.20 | 8 | 52.5 | 0.99196 | 1.0000 |
| Mtnr1b | 1.00 | 0.06 | 8 | 17.5 |  | 0.83 | 0.08 | 8 | 28.4 | 0.15555 | 0.7260 |
| Myod1 | 1.00 | 0.10 | 8 | 28.7 |  | 0.93 | 0.11 | 8 | 33.3 | 0.60124 | 0.8895 |
| Ncoa3 | 1.00 | 0.07 | 8 | 21.2 |  | 0.97 | 0.03 | 8 | 8.3 | 0.84328 | 0.9664 |
| Nfil3 | 1.00 | 0.06 | 8 | 17.5 |  | 0.93 | 0.07 | 8 | 20.4 | 0.44898 | 0.7770 |
| Nkx2-5 | 1.00 | 0.07 | 8 | 20.6 |  | 0.90 | 0.04 | 8 | 11.9 | Unexpressed | |
| Nms | 1.00 | 0.20 | 8 | 56.3 |  | 1.48 | 0.13 | 8 | 24.1 | 0.06834 | 0.6250 |
| Npas2 | 1.00 | 0.07 | 8 | 20.6 |  | 0.90 | 0.04 | 8 | 11.9 | Unexpressed | |
| Nr1d1 | 1.00 | 0.09 | 8 | 24.2 |  | 0.88 | 0.06 | 8 | 19.8 | 0.30487 | 0.7260 |
| Nr1d2 | 1.00 | 0.04 | 8 | 10.7 |  | 0.94 | 0.02 | 8 | 6.5 | 0.22113 | 0.7260 |
| Nr2f6 | 1.00 | 0.09 | 8 | 25.2 |  | 0.93 | 0.05 | 8 | 15.0 | 0.6367 | 0.8950 |
| Opn3 | 1.00 | 0.04 | 8 | 10.7 |  | 0.97 | 0.04 | 8 | 10.6 | 0.61295 | 0.8895 |
| Opn4 | 1.00 | 0.11 | 8 | 30.1 |  | 0.75 | 0.08 | 8 | 29.5 | 0.07172 | 0.6250 |
| Pax4 | 1.00 | 0.10 | 8 | 29.0 |  | 0.90 | 0.07 | 8 | 20.6 | 0.45005 | 0.7770 |
| Per1 | 1.00 | 0.07 | 8 | 20.8 |  | 1.00 | 0.08 | 8 | 23.9 | 0.96707 | 1.0000 |
| Per2 | 1.00 | 0.07 | 8 | 19.0 |  | 1.12 | 0.07 | 8 | 17.2 | 0.24842 | 0.7260 |
| Per3 | 1.00 | 0.04 | 8 | 10.8 |  | 0.98 | 0.06 | 8 | 16.6 | 0.6622 | 0.8954 |
| Pou2f1 | 1.00 | 0.05 | 8 | 15.2 |  | 0.93 | 0.04 | 8 | 11.3 | 0.34559 | 0.7306 |
| Ppara | 1.00 | 0.06 | 8 | 17.2 |  | 1.01 | 0.04 | 8 | 10.9 | 0.85383 | 0.9664 |
| Ppargc1a | 1.00 | 0.06 | 8 | 18.0 |  | 0.80 | 0.03 | 8 | 9.2 | 0.00685 | 0.2985 |
| Prf1 | 1.00 | 0.07 | 8 | 20.3 |  | 1.03 | 0.13 | 8 | 35.4 | Unexpressed | |
| Prkaca | 1.00 | 0.06 | 8 | 17.7 |  | 0.91 | 0.04 | 8 | 13.1 | 0.34195 | 0.7306 |
| Prkacb | 1.00 | 0.04 | 8 | 11.3 |  | 0.93 | 0.05 | 8 | 16.4 | 0.30961 | 0.7260 |
| Prkar1a | 1.00 | 0.03 | 8 | 8.5 |  | 0.95 | 0.02 | 8 | 5.8 | 0.21343 | 0.7260 |
| Prkar1b | 1.00 | 0.06 | 8 | 16.1 |  | 1.02 | 0.03 | 8 | 7.8 | 0.71996 | 0.9259 |
| Prkar2a | 1.00 | 0.02 | 8 | 5.3 |  | 1.00 | 0.02 | 8 | 6.4 | 0.91604 | 0.9979 |
| Prkar2b | 1.00 | 0.04 | 8 | 10.4 |  | 1.02 | 0.03 | 8 | 8.8 | 0.72436 | 0.9259 |
| Prkca | 1.00 | 0.06 | 8 | 17.7 |  | 0.97 | 0.02 | 8 | 5.4 | 0.80119 | 0.9664 |
| Prkcb | 1.00 | 0.08 | 8 | 21.5 |  | 0.87 | 0.02 | 8 | 5.1 | 0.11489 | 0.7260 |
| Prokr2 | 1.00 | 0.11 | 8 | 30.1 |  | 0.92 | 0.10 | 8 | 29.9 | 0.61485 | 0.8895 |
| Ptgds | 1.00 | 0.15 | 8 | 41.6 |  | 1.13 | 0.13 | 8 | 32.6 | 0.49562 | 0.8046 |
| Rora | 1.00 | 0.04 | 8 | 10.2 |  | 0.90 | 0.02 | 8 | 6.2 | 0.02745 | 0.4785 |
| Rorb | 1.00 | 0.06 | 8 | 16.0 |  | 0.92 | 0.04 | 8 | 13.5 | 0.27711 | 0.7260 |
| Rorc | 1.00 | 0.10 | 8 | 28.1 |  | 0.94 | 0.08 | 8 | 23.7 | 0.65003 | 0.8954 |
| Slc9a3 | 1.00 | 0.27 | 8 | 76.3 |  | 1.13 | 0.12 | 8 | 29.9 | 0.30455 | 0.7260 |
| Smad4 | 1.00 | 0.05 | 8 | 13.6 |  | 0.95 | 0.04 | 8 | 10.5 | 0.46364 | 0.7770 |
| Sp1 | 1.00 | 0.05 | 8 | 13.2 |  | 0.95 | 0.03 | 8 | 9.3 | 0.36349 | 0.7316 |
| Srebf1 | 1.00 | 0.14 | 8 | 38.4 |  | 0.92 | 0.12 | 8 | 35.9 | 0.75429 | 0.9259 |
| Stat5a | 1.00 | 0.07 | 8 | 18.9 |  | 0.88 | 0.08 | 8 | 26.2 | 0.24179 | 0.7260 |
| Tcfap2a | 1.00 | 0.32 | 8 | 91.4 |  | 0.63 | 0.06 | 8 | 26.7 | Unexpressed | |
| Tef | 1.00 | 0.05 | 8 | 15.3 |  | 0.93 | 0.04 | 8 | 10.8 | 0.32189 | 0.7260 |
| Tgfb1 | 1.00 | 0.08 | 8 | 22.9 |  | 1.05 | 0.10 | 8 | 26.9 | 0.74784 | 0.9259 |
| Timeless | 1.00 | 0.11 | 8 | 30.4 |  | 1.16 | 0.08 | 8 | 19.3 | 0.20057 | 0.7260 |
| Wee1 | 1.00 | 0.04 | 8 | 10.7 |  | 0.87 | 0.03 | 8 | 11.2 | 0.02648 | 0.4785 |

**Supplementary Figure Legends**

**Figure S1. Eucalyptus smoke exposure elicits changes in heart rate and blood pressure measures.** Minute-to-minute cardiovascular physiology was recorded using implantable telemetry in male and female Sprague Dawley rats to measure cardiovascular parameters prior to (8 hrs; 11:00 AM to 7:00 PM), during (1 hr 9:30 to 10:30 AM), and after (8 hrs; 11:00 AM to 7:00 PM) filtered air or eucalyptus smoke exposure. Data were separated by sex. The following measurements were calculated: heart rate (a) systolic blood pressure (b) diastolic blood pressure (c) pulse pressure (d) and mean arterial pressure (e). Data are mean ± SEM and analyzed by repeated measures two-way ANOVA with Šídák’s post hoc test. Data are reported in 1-min intervals *p < 0.05. n = 8 per group.

**Figure S2. Eucalyptus smoke exposure elicits changes in mean arterial pressure during and after exposure in male rats and decreases in temperature during exposure in female rats.** Cardiovascular physiology was recorded using implantable telemetry to measure cardiovascular parameters and temperature prior to (8 hrs), during (1 hr), and after (8 hrs) filtered air or eucalyptus smoke exposure. Data were separated by sex. The following measurements were calculated: mean arterial pressure during exposure (a) percent change in mean arterial pressure, pre- vs. post-exposure (b) body temperature during exposure (c) and percent change in temperature, pre- vs. post-exposure (d) Data are mean ± SEM and analyzed by repeated measures two-way ANOVA with Šídák’s post hoc test. Data during exposure are reported in 5-min intervals; percent change data after exposure are reported in 1-hr intervals. *p < 0.05. n = 8 per group.

**Figure S3. Eucalyptus smoke exposure and sleep-wake architecture.** EEG telemetry was recorded in male and female Sprague Dawley rats to measure polysomnography parameters, rapid-eye-movement (REM) sleep, non-rapid eye movement (NREM) sleep, and wake prior to (8 hrs; 11:00 AM to 7:00 PM), during (1 hr 9:30 to 10:30 AM), and after (8 hrs; 11:00 AM to 7:00 PM) filtered air or eucalyptus smoke exposure. Data were separated by sex. The following sleep-wake architecture parameters were measured: total NREM bouts during exposure (a) % change in total NREM bouts post-exposure (relative to pre-exposure) (b) total wake bout number during exposure (c) percent change in wake bout number post-exposure (relative to pre-exposure) (d) NREM bout duration during exposure (e) percent change in NREM bout duration post-exposure (relative to pre-exposure) (f) wake bout duration during exposure (g) percent change in wake bout duration post-exposure (relative to pre-exposure) (h) total NREM time during exposure (i) percent change in NREM duration post-exposure (relative to pre-exposure) (j) total wake time during exposure (k) and percent change in total wake duration post-exposure (relative to pre-exposure). Data are mean ± SEM and analyzed by unpaired Student’s t-test. Data during exposure are reported as averages over the ~ 60-min exposure period; percent change data after exposure are reported as averages over the 8-hour post-exposure period. *p < 0.05. n = 8 per group.

**Figure S4. Eucalyptus smoke exposure delays slow wave sleep onset in males.** EEG telemetry was recorded in male and female Sprague Dawley rats to measure polysomnography parameters, rapid-eye-movement (REM) sleep, non-rapid eye movement (NREM) sleep, and wake prior to (8 hrs; 11:00 AM to 7:00 PM), during (1 hr 9:30 to 10:30 AM), and after (8 hrs; 11:00 AM to 7:00 PM) filtered air or eucalyptus smoke exposure. Data were separated by sex. The following sleep parameters were measured: total sleep time during exposure (a) percent change in total sleep time post-exposure (relative to pre-exposure) (b) wake after sleep onset during exposure (c) percent change wake after sleep onset post-exposure (relative to pre-exposure) (d) slow wave sleep onset during exposure (e) slow wave sleep onset post- minus pre-exposure (f) Data are mean ± SEM and analyzed by Student’s unpaired t-test. Data during exposure are reported as averages over the ~ 60-min exposure period; percent change data after exposure are reported as averages over the 8-hour post-exposure period. *p<0.05. n = 8 per group.

**Figure S5. Eucalyptus smoke enhances NREM delta power post-exposure.** EEG telemetry was recorded in Sprague Dawley rats to measure spectral power for rapid-eye-movement (REM) sleep and non-rapid eye movement (NREM) sleep and wake prior to (8 hrs; 11:00 AM to 7:00 PM), during (1 hr 9:30 to 10:30 AM), and after (8 hrs; 11:00 AM to 7:00 PM) filtered air or eucalyptus smoke exposure. Data were separated by sex and analyzed using discrete fast Fourier transform to determine spectral power for REM and NREM. Pre- and post-exposure periods were compared: female REM spectral power (0-20 Hz; a) male REM spectral power (0-20 Hz; b) female REM theta power (4-8 Hz; c) male REM theta power (4-8 Hz; d) female NREM power (0-20 Hz; e) male NREM power (0-20 Hz; f) female NREM delta power (0.5-4 Hz; g) male NREM delta power (0.5-4 Hz, h). Data are mean ± SEM and analyzed by repeated measures two-way repeated measures ANOVA with Šídák’s post hoc test. *p < 0.05, **p < 0.01, ***p < 0.001, ****p < 0.0001. n = 8 per group.

**Figure S6. Eucalyptus smoke reduces alpha power in females.** EEG telemetry was recorded in Sprague Dawley rats to measure spectral power for wakefulness prior to (8 hrs; 11:00 AM to 7:00 PM), during (1 hr 9:30 to 10:30 AM), and after (8 hrs; 11:00 AM to 7:00 PM) filtered air or eucalyptus smoke exposure. Data were separated by sex and analyzed using discrete fast Fourier transform to determine wake spectral power (0.5-20 Hz) for females (a) and males (b). Alpha power (8-12 Hz) was assessed for females (c) and males (d). Data are mean ± SEM and analyzed by repeated measures two-way ANOVA with Šídák’s post hoc test. *p < 0.05, ****p < 0.01. n = 8 per group.

**Figure S7. Eucalyptus smoke exposure had a minimal effect on activity levels.** Blood pressure and EEG Telemetry were recorded in Sprague Dawley rats to measure cardiovascular and polysomnography parameters, respectively, prior to (8 hrs; 11:00 AM to 7:00 PM), during (1 hr 9:30 to 10:30 AM), and after (8 hrs; 11:00 AM to 7:00 PM) filtered air or eucalyptus smoke exposure. Data were separated by sex. The following measurements were calculated: home cage activity during exposure in 5-minute intervals (a) and home cage activity percent change, pre- vs. post-exposure. Data are mean ± SEM analyzed by repeated measures two-way ANOVA with Šídák’s post hoc test. Data during exposure are reported in 5-min intervals; percent change data after exposure are reported in 1-hr intervals..n = 8 per group.

**Figure S8. Activity is negatively correlated with NREM duration across all groups during exposures and before and after eucalyptus smoke (ES) exposures.** EEG telemetry and home cage activity was recorded in male and female Sprague Dawley rats to measure non-rapid-eye movement (NREM) sleep and home cage activity wake prior to (8 hrs; 11:00 AM to 7:00 PM), during (1 hr 9:30 to 10:30 AM), and after (8 hrs; 11:00 AM to 7:00 PM) filtered air or eucalyptus smoke (ES) exposure. A simple linear regression was performed to assess the relationship between home cage activity (y-axis) and NREM duration (x-axis) for exposure periods (1 hour, 5-minute intervals) and before and after exposure (8 hours, 1-hour intervals). Averages were calculated across all animals per treatment group. The following correlations were performed: (a) females, filtered air during exposure (b) females, filtered air pre- and post- exposure (c) females, ES during exposure (d) females, ES pre- and post- exposure (e) males, filtered air during exposure, (f) males, filtered air pre- and post- exposure (g) males, ES during exposure (h) males, ES pre- and post- exposure. Data were analyzed by simple linear regression. n = 8 per group. r^2^ values and significance level are indicated.

**Figure S9. Eucalyptus smoke causes alterations in frequency domain variables of heart rate variability (HRV).** Cardiovascular physiology was recorded using implantable telemetry male and female Sprague Dawley rats to measure cardiovascular parameters prior to (8 hrs; 11:00 AM to 7:00 PM), during (1 hr 9:30 to 10:30 AM), and after (8 hrs; 11:00 AM to 7:00 PM) filtered air or eucalyptus smoke exposure. Data were separated by sex, and exposure data was measured in 5-min intervals while pre- and post-exposure data was measured hourly. The following measurements were calculated for frequency bands related to HRV: normalized high frequency oscillations (nHF) during exposure (a) percent change in nHF, post-exposure (relative to pre-exposure) (b) very low frequency (vLF) oscillations during exposure (c) percent change in vLF, post-exposure (relative to pre-exposure) (d) LF during exposure (e) percent change in LF, post-exposure (relative to pre-exposure) (f) HF during exposure and (g) percent change in HF, post-exposure (relative to pre-exposure). Data are mean ± SEM and analyzed by repeated measures mixed effects analysis with Šídák’s post hoc test. Data during exposure are reported in 5-min intervals; percent change data after exposure are reported in 1-hr intervals. *p < 0.05. n = 8 per group

**Figure S10. Eucalyptus smoke increases standard deviation of diastolic blood pressure during exposure and successive variation of systolic blood pressure after exposure in females.** Cardiovascular physiology was recorded using implantable telemetry in male and female Sprague Dawley rats to measure cardiovascular parameters prior to (8 hrs; 11:00 AM to 7:00 PM), during (1 hr 9:30 to 10:30 AM), and after (8 hrs; 11:00 AM to 7:00 PM) filtered air or eucalyptus smoke exposure. Data were separated by sex. The following measurements were calculated: standard deviation (SD) of systolic blood pressure (BP) during exposure (a) percent change in SD of systolic BP post-exposure (relative to pre-exposure) (b) SD of diastolic BP during exposure (c) percent change in SD post-exposure (relative to pre-exposure) (d) successive variation (SV) of systolic BP during exposure (e) percent change in SV of systolic BP, post-exposure (relative to pre-exposure) (f) SV of diastolic BP during exposure (g) and percent change in SV of diastolic BP, post-exposure (relative to pre-exposure) (h). Data are mean ± SEM and analyzed by Student’s t-test (figures a, c, e. and g), or repeated measures two-way ANOVA with Šídák’s post hoc test (figures b, d, f, and h). Data during exposure are reported as averages during the entire exposure period; percent change data after exposure are reported in 1-hr intervals. *p<0.05. n = 8 per group.

**Supplementary Figures**

**Figure S1**

**Figure S2:**


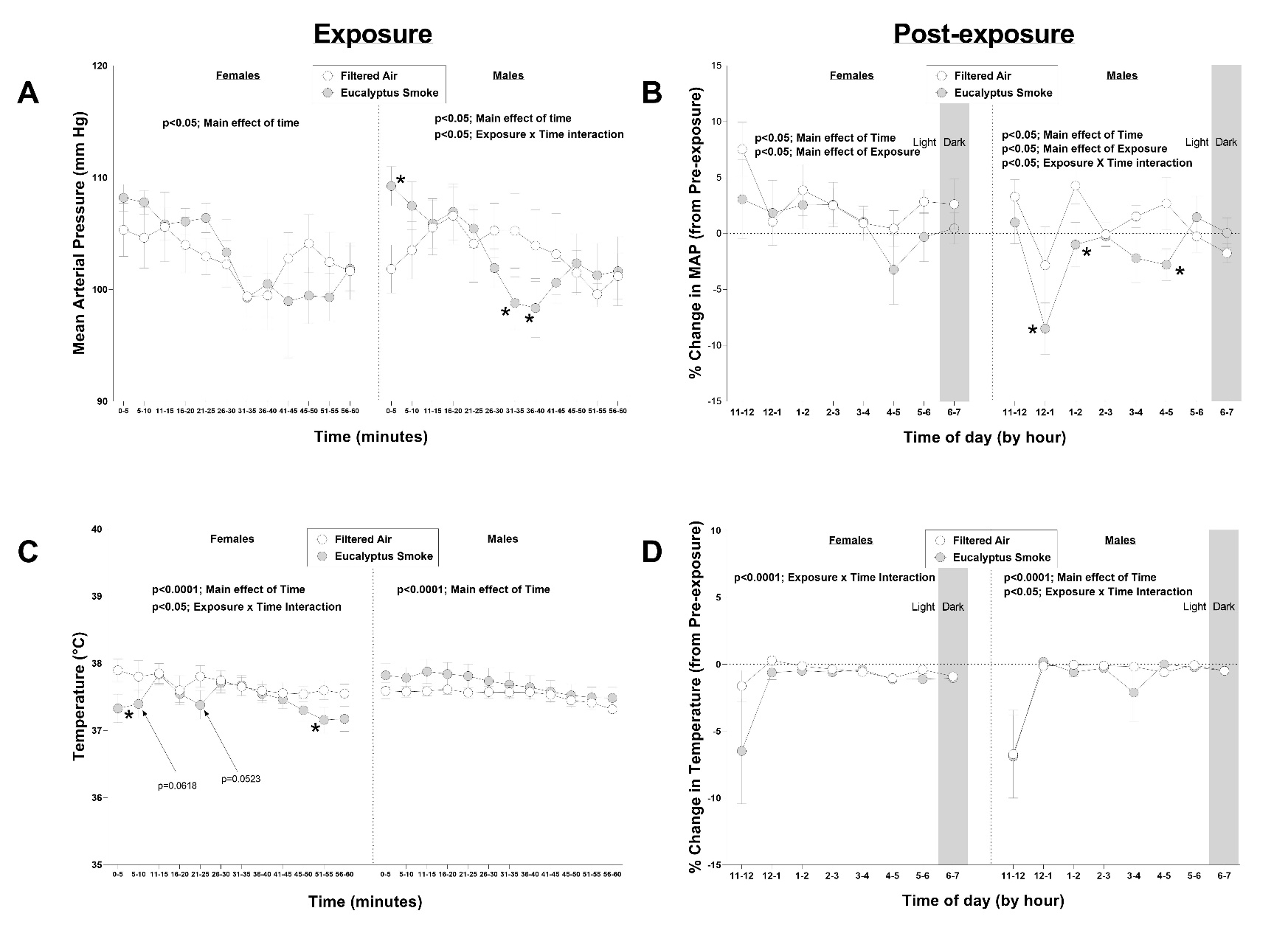


**Figure S3:**

**Figure S4:**

**Figure S5:**

**Figure S6:**

**Figure S7:**

**Figure S8:**

**Figure S9:**

**Figure S10:**
